# Sensitivity of Mitochondrial Transcription and Resistance of RNA Polymerase II Dependent Nuclear Transcription to Antiviral Ribonucleosides

**DOI:** 10.1371/journal.ppat.1003030

**Published:** 2012-11-15

**Authors:** Jamie J. Arnold, Suresh D. Sharma, Joy Y. Feng, Adrian S. Ray, Eric D. Smidansky, Maria L. Kireeva, Aesop Cho, Jason Perry, Jennifer E. Vela, Yeojin Park, Yili Xu, Yang Tian, Darius Babusis, Ona Barauskus, Blake R. Peterson, Averell Gnatt, Mikhail Kashlev, Weidong Zhong, Craig E. Cameron

**Affiliations:** 1 Department of Biochemistry and Molecular Biology, The Pennsylvania State University, University Park, Pennsylvania, United States of America; 2 Gilead Sciences, Inc., Foster City, California, United States of America; 3 Frederick National Laboratory for Cancer Research, NCI, Frederick, Maryland, United States of America; 4 Department of Medicinal Chemistry, The University of Kansas, Lawrence, Kansas, United States of America; 5 Department of Pharmacology, University of Maryland School of Medicine, Baltimore, Maryland, United States of America; Fundación Instituto Leloir-CONICET, Argentina

## Abstract

Ribonucleoside analogues have potential utility as anti-viral, -parasitic, -bacterial and -cancer agents. However, their clinical applications have been limited by off target effects. Development of antiviral ribonucleosides for treatment of hepatitis C virus (HCV) infection has been hampered by appearance of toxicity during clinical trials that evaded detection during preclinical studies. It is well established that the human mitochondrial DNA polymerase is an off target for deoxyribonucleoside reverse transcriptase inhibitors. Here we test the hypothesis that triphosphorylated metabolites of therapeutic ribonucleoside analogues are substrates for cellular RNA polymerases. We have used ribonucleoside analogues with activity against HCV as model compounds for therapeutic ribonucleosides. We have included ribonucleoside analogues containing 2′-C-methyl, 4′-methyl and 4′-azido substituents that are non-obligate chain terminators of the HCV RNA polymerase. We show that all of the anti-HCV ribonucleoside analogues are substrates for human mitochondrial RNA polymerase (POLRMT) and eukaryotic core RNA polymerase II (Pol II) in vitro. Unexpectedly, analogues containing 2′-C-methyl, 4′-methyl and 4′-azido substituents were inhibitors of POLRMT and Pol II. Importantly, the proofreading activity of TFIIS was capable of excising these analogues from Pol II transcripts. Evaluation of transcription in cells confirmed sensitivity of POLRMT to antiviral ribonucleosides, while Pol II remained predominantly refractory. We introduce a parameter termed the mitovir (*mito*chondrial dysfunction caused by anti*vir*al ribonucleoside) score that can be readily obtained during preclinical studies that quantifies the mitochondrial toxicity potential of compounds. We suggest the possibility that patients exhibiting adverse effects during clinical trials may be more susceptible to damage by nucleoside analogs because of defects in mitochondrial or nuclear transcription. The paradigm reported here should facilitate development of ribonucleosides with a lower potential for toxicity.

## Author Summary

Ribonucleoside analogues have potential utility as anti-viral, -parasitic, -bacterial and -cancer agents. However, their clinical applications have been limited by side effects of unknown origin. Here we show in biochemical and cell-based studies that antiviral ribonucleotide analogues are substrates for human mitochondrial RNA polymerase (POLRMT) and eukaryotic core RNA polymerase II (Pol II) in vitro. Analogues that terminate RNA synthesis by viral RNA polymerases also inhibit these cellular RNA polymerase. Importantly, the TFIIS proofreading activity of Pol II is capable of excising these analogues from Pol II transcripts. We introduce a parameter termed the mitovir (*mito*chondrial dysfunction caused by anti*vir*al ribonucleoside) score that can be readily obtained during preclinical studies that quantifies the mitochondrial toxicity potential of compounds. We suggest the possibility that patients exhibiting adverse effects during clinical trials may be more susceptible to damage by nucleoside analogs because of defects in mitochondrial or nuclear transcription. The paradigm reported here should facilitate development of ribonucleosides with a lower potential for toxicity.

## Introduction

Many prokaryotic and eukaryotic organisms have evolved to produce ribonucleoside analogues with antibiotic activity [1]–[3]. Many of these natural products have been shown to have therapeutic efficacy in the treatment of microbial infections and cancer [1]–[3]. These natural therapeutic ribonucleoside analogues have inspired the design of synthetic analogues for a variety of clinical indications [1], [4]–[7].

Hepatitis C virus (HCV) infection is the leading cause of end-stage liver disease and liver cancer in North America and Europe [8]. Among classes of agents being pursued as next generation HCV inhibitors, ribonucleoside analogues that inhibit viral RNA synthesis subsequent to incorporation of their phosphorylated metabolites into nascent viral RNA by the HCV RNA-dependent RNA polymerase [RdRp; HCV non-structural protein 5B (NS5B)] have emerged as perhaps the most promising due to their pangenotype activity and high barrier to resistance development [4], [9]–[11]. After more than a decade, however, not a single anti-HCV ribonucleoside has made it through phase 3 clinical trials due mainly to the manifestation of adverse effects during clinical development.

Promising results for the nucleoside analog MK608 (7-deaza-2′-C-methyladenosine) observed in chimps [12], [13] have never been replicated in the clinic for undisclosed reasons. Particularly frustrating and expensive has been the failure of candidates late in phase 2 studies. The first two nucleoside analogs to enter clinical development, NM283 (prodrug of 2′-C-methylcytosine) and RG1626 (prodrug of 4′-azidocytosine), were stopped due to their respective associations with dose limiting gastrointestinal and hematologic toxicity [4], [10]. PSI-938 (prodrug of 2′-deoxy-2′-fluoro-2′-C-methylguanosine) was also placed on clinical hold following the observation of laboratory abnormalities associated with liver functional tests [14]. Most recently, clinical development of another promising candidate BMS-986094 (prodrug of 2′-C-methylguanosine) was stopped [15]. Many other nucleoside analogs have likely failed during preclinical development due to toxicity.

It has been recognized that many adverse effects associated with nucleoside reverse transcriptase inhibitors (NRTIs), analogues of 2′-deoxynucleosides, used for the treatment of human immunodeficiency virus (HIV) and hepatitis B virus (HBV) are likely precipitated by mitochondrial toxicity [16]–[18]. The potential for severe consequences caused by NRTI induced mitochondrial toxicity was punctuated by the observation of hepatotoxicity during clinical trials of the anti-HBV drug candidate fialuridine [19]. After entering the cell NRTIs and their phosphorylated metabolites partition between the cytoplasm and mitochondria facilitated by nucleoside and nucleotide transporters [16], [20]. In mitochondria, many triphosphorylated NRTIs serve as substrates for the mitochondrial DNA polymerase gamma (Pol γ) [17], [21]. Because mtDNA is present in vast excess of the level that is required to support mitochondrial function, reduction of mtDNA copy number will not manifest in preclinical assays traditionally employed to evaluate compound toxicity [22]. The recognition of Pol γ as an off target of NRTIs therefore required direct approaches, including development of specific biochemical assays to assess the potential of preclinical candidates to elicit mitochondrial dysfunction [17], [21], [23].

mtDNA encodes 13 proteins required for production of ATP by oxidative phosphorylation, as well as all of the rRNAs and tRNAs required for translation within mitochondria of the corresponding 13 mRNAs. Therefore, reduction of mtDNA gene expression can lead to mitochondrial dysfunction [24]. Evaluation of antiviral ribonucleoside toxicity in vitro typically measures the effect of a compound on the intracellular concentration of ATP [25]. The ATP level clearly provides an indication of the energy status of the cell and is thought to be a useful surrogate for mitochondrial (dys)function. To date, not a single study has evaluated directly the impact of antiviral ribonucleoside treatment of cells on mitochondrial transcription. When assessed, nuclear transcription has often been ruled out as an off target for antiviral ribonucleotides [26], [27].

Here we present biochemical and cellular evidence that antiviral ribonucleoside triphosphates are substrates for the human mitochondrial RNA polymerase (POLRMT) as well as the core polymerase of eukaryotic RNA polymerase II (Pol II). In our analysis, we included analogues containing substituents present in several anti-HCV lead compounds that have been termed non-obligate chain terminators because they contain a 3′-OH group yet prevent elongation of nascent viral RNA. To our surprise, all of the non-obligate chain terminators inhibited RNA elongation by POLRMT and Pol II as well. The addition of transcription factor S-II (TFIIS) to Pol II reactions reduced substantially the level of analogue incorporated, thus explaining the relatively reduced effect of the analogues on Pol II-dependent transcription in cells. The efficiency of incorporation by POLRMT in vitro predicted outcomes in cells when normalized for intracellular metabolism of the antiviral ribonucleoside to the triphosphorylated form.

We present evidence that attrition during clinical studies of ribonucleosides with anti-HCV activity may be a reflection of inhibited mitochondrial gene expression. The potential for these effects is likely missed during in vitro studies completed in cell culture because traditional assays only evaluate ATP output. Early clinical studies may not detect adverse events because a sufficient number of patients are not exposed to identify those most sensitive to mitochondrial toxicity. Mitochondrial toxicity has been characterized as a threshold effect [22], [24] and duration of the insult and the genetic predisposition of patients may be important factors in the severity of adverse events observed. We propose that genetic determinants of sensitivity to therapeutic nucleosides will not only include pathways required for mitochondrial function but also pathways that contribute to the fidelity of nuclear transcription.

## Results

### Antiviral ribonucleoside triphosphates are substrates for POLRMT

Development of antiviral ribonucleosides targeting HCV NS5B has been thwarted by the tendency for many lead compounds to elicit adverse effects either in Investigational New Drug (IND)-enabling animal studies or, worse, in patients in the clinic [4], [10]. It is becoming increasingly clear that late-stage attrition of drug candidates is often caused by off targets affecting mitochondrial function [28]. Moreover, it is well documented that the triphosphorylated metabolites of nucleoside reverse transcriptase inhibitors (NRTIs) are substrates for Pol γ, leading to reduced mitochondrial DNA copy number and eventually mitochondrial dysfunction [17], [21], [23].

**Figure 1:**
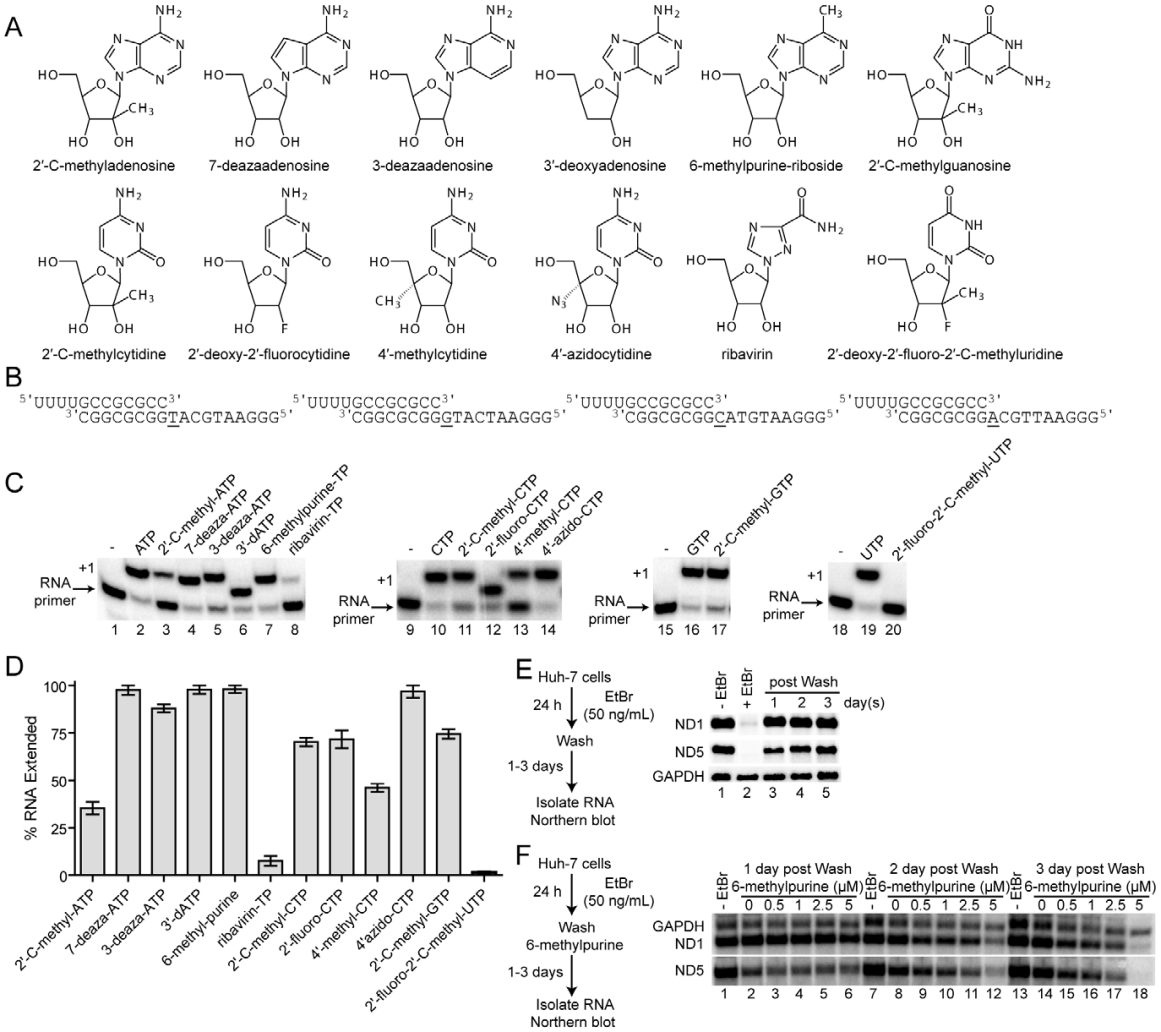
Antiviral ribonucleoside triphosphates are substrates for POLRMT. (**A**) Nucleoside analogs used in this study. (**B**) RNA-primed DNA templates. The first templating base is underlined. (**C**) Nucleoside analog incorporation catalyzed by POLRMT. Reaction products from POLRMT-catalyzed nucleotide incorporation using the indicated nucleoside analog triphosphate and RNA primer/DNA template nucleic acid scaffolds shown in panel b. Reactions proceeded for 30 s. RNA primer was extended to n+1 in the presence of each nucleoside triphosphate. (**D**) Percentage of RNA product relative to correct nucleotide (ATP, CTP, GTP or UTP) is shown. Error bars represent s.e.m. n = 3. Both 2′-deoxy-2′-fluoro-2′-C-methyl-UTP and ribavirin triphosphate were the least incorporated nucleoside analogs. (**E**) Inhibition of mitochondrial transcription by ethidium bromide (EtBr) and the recovery of mitochondrial transcription. Northern blots of mitochondrial transcripts ND1, ND5 and nuclear transcript GAPDH 24 h post EtBr treatment and 1, 2 and 3 day(s) post wash. Mitochondrial transcripts ND1 and ND5 were inhibited by EtBr treatment and recovered post wash; there was no effect on GAPDH. (**F**) Effect of 6-methlypurine on POLRMT-catalyzed transcription in vivo. Northern blots of ND1, ND5 and GAPDH after EtBr treatment and recovery in the presence of 6-methylpurine.

We recently developed the biochemical tools to study substrate utilization by POLRMT in vitro, placing us in a unique position to determine the extent to which utilization of antiviral ribonucleoside triphosphates by POLRMT may contribute to attrition [29], [30]. For this study, we selected the panel of purine and pyrimidine analogues shown in **Figure 1A**. This panel contains modifications to the base or ribose found in past and/or current clinical candidates for treatment of HCV [4], [9]–[11], [31]–[35]. Ribavirin was included to set the baseline for “tolerable” values of any observed off-target activity. All of these analogues were converted to the triphosphate in cells; however, the steady-state levels varied from less than 0.15 µM to 3.5 mM (**Table 1**). For 2′-C-methyladenosine, we also determined the concentration of the triphosphate in isolated mitochondria. This value was 210 µM (**Table S1**), a value in the range determined when total cellular pools were evaluated (**Table 1**). All of the ribonucleosides except 4′-methylcytidine inhibited replication (**Table 1**, **EC_50_**) of HCV in the human hepatoma cell line, Huh-7, at a concentration that was, in most cases, below the value of ribonucleoside required to reduce the intracellular ATP levels by 50% (CC_50_) as measured by a luciferase reporter assay (**Table 1**).

**Table 1:** Intracellular metabolism, cytotoxicity (CC_50_), anti-HCV replicon activity (EC_50_) and anti-NS5B activity (IC_50_)^a^.

Ribonucleoside	[TP] (µM)^b^		CC_50_ (µM)		EC_50_ (µM)	IC_50_ (µM)
	Huh-7	MT4	Huh-7	MT4	Huh-7 1b	NS5B
			(5 day)	(5 day)	replicon cells	
2′-C-methyladenosine	620±20	120±10	65±22	6±1	0.47±0.04	8.0±2.3
7-deazaadenosine	3500±10	1200±200^c^	0.046±0.027	0.023±0.010	0.0087±0.04	>200
3-deazaadenosine	1.0±0.05	0.55±0.02	190	110±10	4.0±1.0	>200
3′-deoxyadenosine	<0.15±0.05	<0.2±0.05	150±10	66±7	29±13	72±14
6-methylpurine-riboside	1000±45	2400±1100^c^	0.10	0.003±0.001	0.075±0.010	nd^d^
ribavirin	30±3	24±2	40±4	40±5	36±14	>1000
2′-C-methylguanosine	3.4±0.33	0.56±0.24	>100	>200	3.3±0.9	0.32±0.15
2′-C-methylcytidine	6.5±2.5	22±3	770±30	20±2	2.3±0.1	0.47±0.16
2′-deoxy-2′-fluorocytidine	21±3	43±9^c^	46±17	4±1	46±22	>200
4′-methylcytidine	0.50±0.03	0.17±0.03	>1000	>1000	>90	7.8±6.7
4′-azidocytidine	2.60±0.20	1.0±0.10	>1000	460±60	12±2	2.9±0.4
2′-deoxy-2′-fluoro- 2′-C-methyluridine^e^	250±20	nd^d^	66±20	>100	0.15±0.03	3.3±0.4

a Values rounded to two significant figures. All values are the mean ± s.d. of at least 3 independent experiments done in duplicate or triplicate except for CC_50_ Huh-7 for 3-deazaadenosine and 6-methylpurine-riboside, which are the average of replicate wells from one experiment.

b Intracellular metabolism [TP] is the amount of nucleoside triphosphate determined from LC/MS/MS analysis and converted from pmol per million cells to intracellular concentration (µM) using a cellular volume of 2 pL per cell. All data for 10 µM 24 h incubations except where noted otherwise.

c Compounds that showed toxicity in MT4 cells at 10 µM. Incubations were done at 0.1 µM and the intracellular levels dose normalized assuming proportional increase in intracellular metabolites with extracellular concentrations.

d not determined.

e Tested in the form of monophosphate prodrug GS-7977.

Utilization of the antiviral ribonucleoside triphosphates by POLRMT was determined by using RNA-primed DNA templates containing the complementary base residue as the first templating base (**Figure 1B**). Initial assays determined the fraction of primer extended after a 30 s incubation of POLRMT in the presence of each nucleotide substrate at a concentration of 500 µM normalized to correct nucleotide utilization (**Figure 1C and D**). Under these conditions, all of the antiviral analogues tested except for 2′-deoxy-2′-fluoro-2′-C-methyluridine were incorporated much more efficiently than ribavirin (compare all lanes to lane 8 of **Figure 1C and D**). This initial screen clearly indicates that POLRMT nucleotide substrate specificity is quite relaxed with respect to non-natural functional groups.

In order to more accurately and quantitatively compare the efficiency of incorporation for each analogue, we evaluated the kinetics of incorporation as a function of analogue concentration and used these data to determine the maximal rate constant for incorporation (*k_pol_*) and apparent dissociation constant (*K_d,app_*) (**Figures S1, S2 and S3**). These values are reported in **Table 2** for all of the analogues. The incorporation efficiency (*k_pol_*/*K_d,app_*) of each analogue was less than that of the correct nucleotide by at least an order of magnitude (**Table 2**). Only one analogue (2′-deoxy-2′-fluoro-2′-C-methyluridine) was incorporated with an efficiency less than ribavirin (**Table 2** **, Figure S2 and S3**). Interestingly, the efficiency of incorporation of the second most inefficient analogue (2′-C-methyl-ATP) was still on the order of 10-fold higher than observed for ribavirin triphosphate (**Table 2** **and Figure S1**).

**Table 2:** Kinetic parameters for POLRMT-catalyzed nucleotide incorporation^a^.

Ribonucleoside triphosphate	k_pol_	K_d,app_	k_pol_/K_d,app_	mitovir score^b^^,^ c	mitovir score^b^^,^ d
	(s^−1^)	(µM)	(µM^−1^ s^−1^)		
ATP (correct)	30±2	20±2	1.5±0.3	30^e^	30^e^
2′-C-methyl-ATP	0.030±0.010	530±100	5.7±2.2×10^−5^	1.7×10^−2^	7.1×10^−3^
7-deaza-ATP	15±2	80±20	1.9±0.5×10^−1^	15	14
3-deaza-ATP	0.10±0.01	340±40	2.9±0.5×10^−4^	3.1×10^−4^	1.6×10^−4^
3′-dATP	0.70±0.02	8.0±1.0	8.8±1.0×10^−2^	1.3×10^−2^	1.7×10^−2^
6-methylpurine-TP	30±5	280±10	1.1±0.2×10^−1^	23	27
ribavirin-TP	0.0050±0.0010	800±100	6.3±1.5×10^−6^	1.8×10^−4^	1.4×10^−4^
2′-dATP	0.20±0.01	700±100	2.9±0.4×10^−4^	8.5×10^−4^	8.5×10^−4^
GTP (incorrect)	0.020±0.002	1100±200	1.8±0.4×10^−5^	9.1×10^−3^	9.1×10^−3^
GTP (correct)	21±1	5.5±0.5	3.8±0.4	21^e^	21^e^
2′-C-methyl-GTP	0.070±0.010	370±130	1.9±0.7×10^−4^	6.4×10^−4^	1.1×10^−4^
CTP (correct)	25±1	20±5	1.3±0.3	23^e^	23^e^
2′-C-methyl-CTP	0.060±0.010	180±20	3.3±0.7×10^−4^	2.1×10^−3^	6.4×10^−3^
2′-deoxy-2′-fluoro-CTP	0.050±0.010	130±30	3.8±1.2×10^−4^	7.0×10^−3^	1.2×10^−2^
4′-methyl-CTP	0.045±0.010	580±40	7.8±1.8×10^−5^	3.9×10^−5^	1.3×10^−5^
4′-azido-CTP	5.1±1.0	580±230	8.8±4.0×10^−3^	2.3×10^−2^	8.8×10^−3^
UTP (correct)	17±1	29±2	0.60±0.05	17^e^	17^e^
2′-deoxy-2′-fluoro- 2′-C-methyl-UTP	3.4±0.6×10^−4^	550±260	6.2±3.0×10^−7^	1.0×10^−4^	nd^f^

a Values rounded to two significant figures. Standard errors are from non-linear regression fits of data to a hyperbolic model (**Figures S1, S2 and S3**).

b *mitovir score*: rate constant for incorporation calculated by using the experimentally determined kinetic parameters, *k*_pol_ and *K*_d,app_ and the intracellular concentration of nucleoside analog triphosphate [TP] (**Table 1**); *mitovir score* = *k*_eff_ (s^−1^) = (*k_pol_* * [TP])/(*K_d,app_*+[TP]).

c *mitovir score* determined for Huh-7 cells.

d *mitovir score* determined for MT4 cells.

e rate constant for incorporation calculated by using the experimentally determined kinetic parameters, *k*_pol_ and *K*_d,app_ and the intracellular concentration of nucleotide [TP] (**Table S1**); *k*_eff_ (s^−1^) = (*k_pol_* * [TP])/(*K_d,app_*+[TP]).

f not determined.

Next, we determined the effect of 6-methylpurine, one of the more efficiently-utilized nucleoside substrate analogues, on mitochondrial transcription in Huh-7 cells (**Figure 1E and F**). We chose to evaluate this analogue because it is the only one that will change the basepairing properties of the base if incorporated and perhaps alter the structure, stability and/or function of mitochondrial RNA. Briefly, mitochondrial transcription was inhibited by treatment of cells with ethidium bromide (EtBr, 50 ng/mL). Levels of two mitochondrial transcripts (ND1 and ND5) were monitored. Treatment of cells with EtBr for 24 h was sufficient to reduce both transcripts to near background levels (ND1 and ND5 in lane +EtBr of **Figure 1E**). This treatment had no effect on transcription by RNA polymerase II (compare GAPDH to ND1 or ND5 in lane +EtBr of **Figure 1E**). When 6-methylpurine was added to media after removal of EtBr, the steady state was reestablished to a level equivalent to that observed when no 6-methylpurine was present (**Figure 1F**). However, after prolonged treatment (3 days) with 6-methylpurine both ND1 and ND5 mitochondrial transcripts were reduced, presumably due to the incorporation of 6-methylpurine and the instability of these transcripts in cells. GAPDH also showed some sensitivity to treatment with 6-methlypurine. Although this result does not permit us to determine whether or not 6-methylpurine is incorporated, it clearly shows a discernible phenotype on the steady-state level of mitochondrial RNA. This circumstance makes it impossible to use analysis of RNA in cells to rule in or out the incorporation of an antiviral ribonucleoside that does not inhibit elongation.

### Non-obligate chain terminators inhibit RNA elongation by POLRMT

The finding that all of the antiviral ribonucleoside triphosphates tested were incorporated by POLRMT was quite surprising. As suggested above, the lack of extreme modifications to the bases may preclude changes to RNA elongation. Several of the most exciting analogues of past and current anti-HCV ribonucleosides contain modifications to the 2′ and/or 4′ position of the ribose ring (e.g. 2′-C-methyladenosine, 2′-C-methylcytidine, 2′-C-methylguanosine and 4′-azidocytidine in **Figure 1A**) [4], [9]–[11]. These modifications may prevent the RdRp from translocating after analogue incorporation or prevent the appropriate positioning of the incoming nucleotide in the active site, thus leading to premature termination of the viral RNA [4], [9]–[11]. These non-obligate chain terminators are therefore quite potent as only one incorporation event per transcript is required to express the inhibitory activity. This is illustrated in the finding that these compounds can inhibit HCV NS5B polymerase activity in vitro quite effectively (**Table 1** **, IC_50_**)

**Figure 2:**
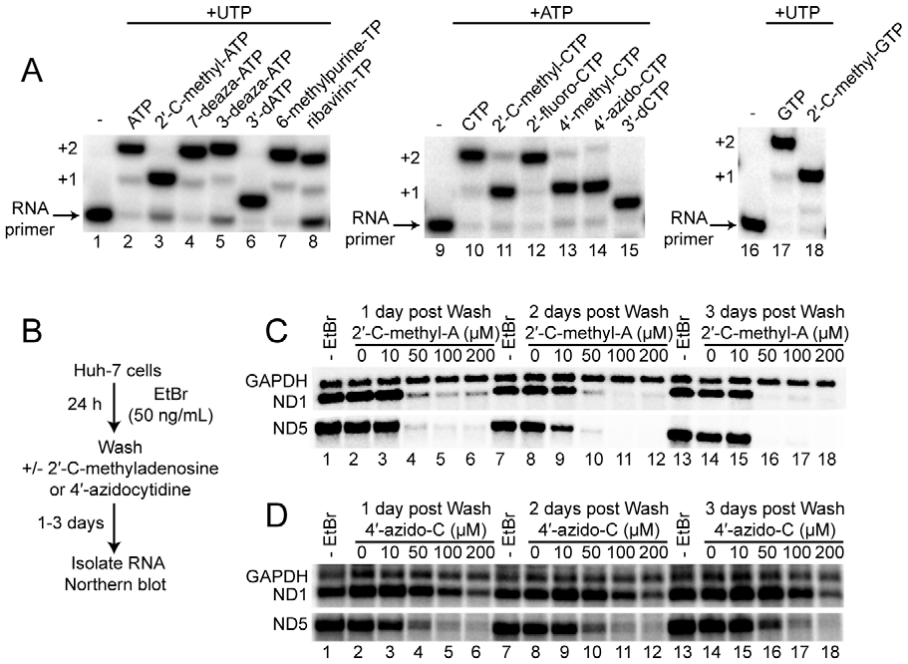
Non-obligate chain terminators inhibit RNA elongation by POLRMT. (**A**) Non-obligate chain termination of RNA synthesis in vitro. Products from POLRMT-catalyzed nucleotide incorporation in the presence of the next correct nucleotide substrate, UTP or ATP. Reactions proceeded for 10 min. Reactions containing ATP, 7-deaza-ATP, 3-deaza-ATP, 6-methylpurine-TP, ribavirin-TP, CTP,2′-deoxy-2′-fluoro-CTP and GTP were readily extended to n+2 product by POLRMT. Reactions containing 2′-C-methyl-ATP, 2′-C-methyl-CTP, 4′-methyl-CTP, 4′-azido-CTP and 2′-C-methyl-GTP were unable to be extended to n+2, demonstrating the ability of these nucleoside analogs to be non-obligate chain terminators for POLRMT once incorporated in nascent RNA. 3′-dATP and 3′-dCTP were used as positive controls. (**B–D**) Production of full-length mitochondrial RNA transcripts in cells is impaired in the presence of 2′-C-methyladenosine and 4′-azidocytidine. (**B**) Experimental design. Huh-7 cells were treated with EtBr for 24 h to deplete mitochondrial transcripts from cells, washed, treated with 2′-C-methyladenosine or 4′-azidocytidine for 1, 2 and 3 days, total RNA isolated and Northern blots performed. Northern blots of ND1, ND5 and GAPDH after EtBr treatment and recovery in the presence of (**C**) 2′-C-methyladenosine and (**D**) 4′-azidocytidine. Cells treated with a minimum of 50 µM 2′-C-methyladenosine showed specific inhibition of mitochondrial transcription and the inability to produce both ND1 and ND5 transcripts, whereas a minimum of 50 µM 4′-azidocytidine only inhibited production of ND5; GAPDH was unaffected by treatment with 50 µM 2′-C-methyladenosine or 4′-azidocytidine. At higher concentrations of 4′-azidocytidine GAPDH showed some sensitivity.

We used the primer extension assay to determine if modifications to the 2′ or 4′ positions of the ribose caused defects to POLRMT elongation. Using the template with thymine as the first templating base and adenine as the second templating base, the combination of ATP and UTP led to extension of the RNA primer by two nucleotides (lane 2 in **Figure 2A**). As a control for chain termination, we used the combination of 3′-dATP, an obligate chain terminator, and UTP. As expected, the RNA primer was extended by only one nucleotide (lane 6 in **Figure 2A**). Surprisingly, the combination of 2′-C-methyl-ATP and UTP produced only the +1 extension product, consistent with this analogue being a non-obligate chain terminator of POLRMT (lane 3 in **Figure 2A**).

We performed the complementary experiment for CTP and analogues thereof using the template with guanine as the first templating base and thymine as the second templating base (**Figure 2A**). The 2′-C-methyl, 4′-methyl and 4′-azido modifications caused substantial inhibition of RNA primer elongation (lanes 11, 13 and 14, respectively, in **Figure 2A**). The 2′-deoxy-2′-fluoro modification did not inhibit elongation (lane 12 in **Figure 2A**). Lastly, the analogue 2′-C-methyl-GTP was also a non-obligate chain terminator of POLRMT (lane 18 in **Figure 2A**)

The availability of nucleosides with chain termination activity provides the opportunity to determine unambiguously the potential for the biochemical experiments to predict effects on mitochondrial transcription in cells. Mitochondrial transcription was inhibited for 24 h using EtBr. EtBr was removed and replaced with media lacking nucleoside or containing 2′-C-methyladenosine (**Figure 2C**) or 4′-azidocytidine (**Figure 2D**). Total RNA was isolated and processed for analysis by northern blotting using probes for mitochondrial RNAs (ND1 and ND5) and a cellular RNA control (GAPDH). We observed a dose-dependent reduction in the abundance of mitochondrial RNAs using both adenosine and cytidine analogues (**Figure 2C and D**). The potency of 2′-C-methyladenosine was greater than that of 4′-azidocytidine. At a concentration of 50 µM, 2′-C-methyladenosine completely inhibited accumulation of both ND1 and ND5 transcripts (lanes 4, 10 and 16 in **Figure 2C**). In contrast, 50 µM 4′-azidocytidine was only sufficient to block ND5 accumulation (compare ND1 and ND5 in lanes 6, 12 and 18 in **Figure 2D**). ND1 is the first cistron and ND5 the last cistron of a near 16,000 nt polycistronic pre-mRNA produced in mitochondria [36]. The content of adenylate and cytidylate residues is equivalent for both ND1 and ND5. The polar effect of 4′-azidocytidine inhibition likely reflects the fact that the probability of incorporation increases as a function transcript length. Thus, genes near the 5′ end will be less susceptible to termination than genes near the 3′ end. That 2′-C-methyladenosine promotes termination becomes even more convincing by evaluating overexposed blots, which show a ladder of products that hybridize to the probe only in drug-treated lanes (**Figure S4**). Collectively, these data demonstrate that biochemical analysis, which includes in vitro POLRMT-catalyzed nucleotide incorporation assays that assess the efficiency of utilization and inhibition, can predict biological outcomes, thus permitting us to conclude that inhibition of POLRMT by antiviral ribonucleosides must be considered during development of this class of antiviral agents.

### TFIIS prevents inhibition of Pol II transcription by non-obligate chain terminators

**Figure 3:**
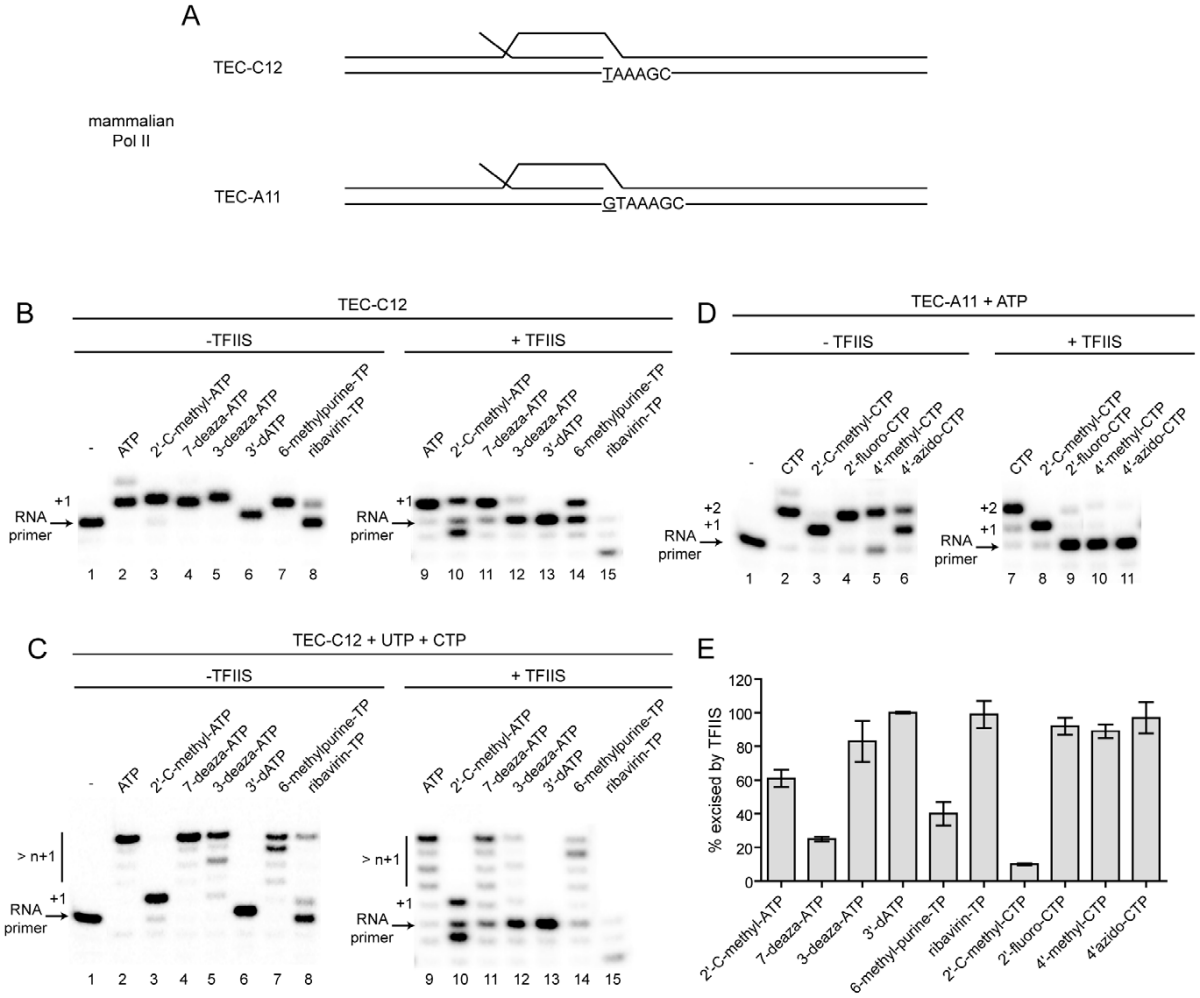
TFIIS prevents accumulation of antiviral nucleotides in Pol II transcripts. (**A**) Schematic of synthetic nucleic scaffolds for transcription elongation complex (TEC) assembly with calf thymus Pol II. The first templating base is underlined. The TEC with 11-nt RNA (TEC-A11) was assembled using TDS50, NDS50 and RNA9 (see **Table S2** for the complete oligonucleotide sequences) in the presence of 10 µM each GTP and ATP (TEC-A11) and purified from the unincorporated DNA, RNA and NTPs; TEC-C12 was obtained by addition of 10 µM CTP to TEC-A11. (**B**) Reaction products from Pol II-catalyzed nucleotide incorporation in the absence and presence of TFIIS. The concentration of unmodified substrate NTP and analogs were 500 µM; TFIIS was added at 10 µM. Reactions proceeded for 2 min. Reactions with ribavirin-TP proceeded for 10 min. (**C,D**) Reaction products from Pol II-catalyzed nucleotide incorporation in the presence of the next correct nucleotide substrate. The concentration of the unmodified substrate NTP and analogs were 500 µM; TFIIS was added at 10 µM. Reactions proceeded for 2 min. Reactions with ribavirin-TP, 4′-methyl-CTP and 4′-azido-CTP proceeded for 10 min. (**E**) Percent inhibition by TFIIS on Pol II nucleoside analog incorporation.

The inability to observe perturbations in the levels of cellular RNAs derived from nuclear transcription in response to antiviral ribonucleoside treatment has been interpreted to mean that nuclear RNA polymerases are unable to use these agents [13], [26]. The observation that neither 2′-C-methyladenosine nor 4′-azidocytidine had a marked effect on the nuclear transcript GAPDH (**Figure 2C and D**) is consistent with these previous studies. In order to test this possibility directly, we used transcription elongation complexes with Pol II isolated from calf thymus and scaffolds capable of templating adenosine analogues (C12 in **Figure 3A**) or cytidine analogues (A11 in **Figure 3A**) as the first nucleotide [37], [38]. With the exception of ribavirin triphosphate (RTP), all of the purine nucleotide analogues were efficiently incorporated by Pol II when the proofreading activity of TFIIS was absent (lanes 1–8 in **Figure 3B**). The presence of TFIIS inhibited accumulation of the analogues in RNA (lanes 9–15 in **Figure 3B**). In order to determine the consequence of analogue incorporation on elongation, the elongation reaction was performed in the presence of purine analogue and the next two nucleotides, UTP and CTP (**Figure 3C**). With the exception of 2′-C-methyl-ATP, all analogues with a 3′-OH supported extension of the RNA to the n+4 position in the absence of TFIIS (lanes 2–6 in **Figure 3C**). Worth noting, RMP incorporation failed to support robust extension (lane 8 in **Figure 3C**). We conclude that non-obligate chain terminators of HCV RdRp have the same impact on Pol II. Again, the presence of TFIIS antagonized production of fully elongated RNA (panel +TFIIS in **Figure 3C**). Interestingly, the observation that TFIIS only partially reverses the effects of 6-methlypurine utilization (**Figure 3B and C**) is consistent with the sensitivity of GAPDH transcripts in cells when treated with this nucleoside (**Figure 1F**).

We performed an analogous set of experiments with the cytidine analogues known to exhibit non-obligate chain termination activity with HCV RdRp (**Figure 3D**). Unexpectedly, the 2′-fluoro, 4′-methyl and 4′-azido modifications permitted elongation by Pol II in the absence of TFIIS. TFIIS efficiently prevented their incorporation and/or extension (**Figure 3D**). Curiously, even though the 2′-C-methyl modification prevented further elongation, TFIIS did not remove the analogue on the timescale of the experiment (lanes 1 and 7 of **Figure 3D**).

We quantified the ability of TFIIS to prevent accumulation of the ribonucleoside analogues in RNA (**Figure 3E**). Two classes of analogues were identified: those that were excised by TFIIS 80% or better and those that were not. Reduced excision of the analogues containing a 2′-C-methyl substituent was a kinetic phenomenon as longer incubations led to greater excision (**Figure S5**). The efficiency with which TFIIS functions to excise ribonucleoside analogues extends to that from *Saccharomyces cerevisiae* (**Figure S6**).

### Predicting adverse effects of antiviral ribonucleosides during preclinical development: Use of MT4 cells and the mitovir score

**Figure 4:**
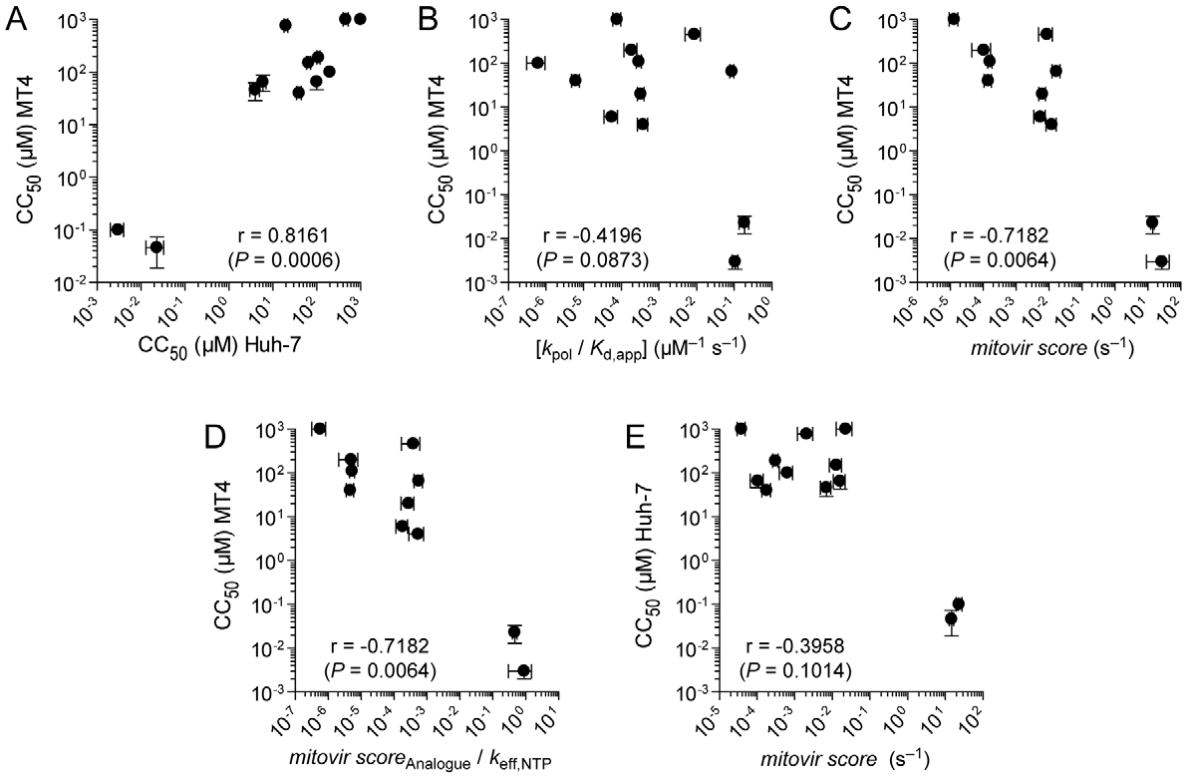
Predicting adverse effects of antiviral ribonucleosides during preclinical development: The mitovir score. Correlations between (**A**) cytotoxicity in Huh-7 cells and MT4 cells (**Table 1** **, CC_50_**), (**B**) cytotoxicity in MT4 cells (**Table 1** **, CC_50_**) and the efficiency of nucleotide incorporation (**Table 2** **,** ***k*** **_pol_/** ***K*** **_d,app_**), (**C**) cytotoxicity in MT4 cells (**Table 1** **, CC_50_**) and *mitovir score* for MT4 cells (**Table 2**), (**D**) cytotoxicity in MT4 cells (**Table 1** **, CC_50_**) and the *mitovir score* for each analogue corrected to account for the presence of the nucleotide with which the analogue competes, ATP or CTP, and (**E**) cytotoxicity in Huh-7 cells (**Table 1** **, CC_50_**) and *mitovir score* for Huh-7 cells (**Table 2**). Error bars represent s.d. Nonparametric (Spearman) correlations with r values shown. In parentheses are one-tailed P-values calculated from Spearman coefficients to provide a measure of statistical significance of correlation.

Why are the adverse effects of antiviral ribonucleoside analogues not recognized sooner? Validated cell model system(s) capable of revealing the adverse effects are lacking. For this study, we have employed three cell lines, with Huh-7 and MT4 cells being the workhorses. The nucleoside analogue triphosphate pools were equivalent in these cell lines (**Table 1**). Metabolism of the antiviral ribonucleosides tested here to the triphosphate were within 2- to 3-fold of each other (**Table 1**). In addition, levels of cytotoxicity caused by the analogues as reported by CC_50_ values exhibited correlation between the MT4 and Huh-7 cell lines r = 0.8161, **Figure 4A**). In general, MT4 cells were an order of magnitude more sensitive to the antiviral nucleosides than Huh-7 cells (**Table 1**). The cytotoxicity observed in MT4 cells was only weakly correlated with the intrinsic efficiency (*k_pol_*/*K_d,app_*) with which POLRMT incorporated the analogue r = −0.4196, **Figure 4B**). However, when the rate constant for incorporation was calculated using the intracellular concentration of the triphosphorylated form of the analogue (**Table 2**, *mitovir score*), this parameter was strongly correlated with the observed cytotoxicity in MT4 cells (r = −0.7182, **Figure 4C**). We refer to this constant, the rate constant for analogue incorporation by POLRMT adjusted for intracellular concentration of the triphosphorylated form of the analogue, as the mitovir score as it provides a prediction of POLRMT-mediated *mito*chondrial dysfunction caused by the anti*vir*al ribonucleoside. Normalizing the mitovir score to account for the presence of the nucleotide with which the analogue competes did not further strengthen the correlation (*P* = 0.0064, **Figure 4D**). The correlation between cytotoxicity and the mitovir score was weaker in Huh-7 cells (r = −0.3958, **Figure 4E**) a less robust cell line for cytotoxicity studies than the MT4 line, yet a correlation is discernible and incorporation of the analogues into mitochondrial RNA clearly occurs (**Figures 2C and 2D**). We suggest that the use of the MT4 cell line in combination with the mitovir score, which represents the combination of biochemical analysis and intracellular metabolism, will be sufficient to identify antiviral ribonucleosides during preclinical development with the potential to cause adverse effects.

## Discussion

Maintenance, replication and expression of the mitochondrial genome (mtDNA) is absolutely essential for cell function because mtDNA encodes 13 proteins required for oxidative phosphorylation, as well as the tRNAs and rRNAs essential to their production [36]. However, cells have far more mtDNA than required to support oxidative phosphorylation; therefore, a substantial reduction in mtDNA copy number is required for manifestation of a clinically apparent phenotype [22], [24]. Therefore, adverse effects of drugs that interfere with replication and/or expression of mtDNA may not be readily apparent in mammalian cells or animal models on the timescale of preclinical testing [28]. In addition, because mammalian cell lines are often grown in high concentrations of glucose, oxidative phosphorylation is not necessarily required for ATP production in cells, the so called Crabtree effect [39]. Under these conditions, even severe mitochondrial dysfunction would have little to no impact on intracellular ATP levels.

A high proportion of anti-HCV ribonucleosides have entered the clinic just to have development discontinued due to adverse effects in patients [4], [10]. The molecular bases for these effects are either not known or not reported. We have shown here for the first time that antiviral ribonucleoside triphosphates are substrates for POLRMT both in vitro and in cells (**Figures 1** **and** **2** **and** **Table 2**), suggesting that patient toxicity in the clinic may be related to mitochondrial dysfunction. That a mitochondrial polymerase is an off target for an antiviral agent is not surprising as this problem has been well documented for Pol γ, resulting from nucleoside NRTIs used in the treatment of HIV infection [17], [21], [23]. What is surprising, however, is the absence of any reported attempts to evaluate POLRMT.

POLRMT is a single-subunit RNA polymerase that is a member of the bacteriophage T7 family of RNA polymerases. Only recently has any mechanistic [30] or structural [40] information become available for this enzyme. The active sites of POLRMT and T7 RNA polymerase (T7 RNAP) differ by only one amino acid [40]. Interestingly, POLRMT can incorporate ribavirin (**Table 2**), but T7 RNAP fails to utilize this antiviral ribonucleotide during in vitro transcription reactions [41]. Checkpoints for incorporation fidelity by POLRMT discovered to date reside at the active site and represent conformational changes observed for all classes of nucleic acid polymerases [30]. The observed differences in fidelity between POLRMT and T7 RNAP may suggest that remote sites may be controlling fidelity [30], thus complicating the use of homology models for the identification of nucleotide modifications that will prevent utilization by POLRMT.

Our biochemical assays are able to predict outcomes in cells when non-obligate chain terminators are used (**Figure 2**). This observation suggests that mitochondrial factors do not exist to cleave the nascent RNA associated with a stalled elongation complex that would be analogous to Gre and TFIIS factors of multi-subunit RNA polymerases [42]. We conclude that the only determinant of the incorporation fidelity observed in cells other than the intrinsic efficiency with which POLRMT utilizes the antiviral ribonucleotide is the size of the intracellular antiviral ribonucleoside triphosphate (rNTP) pool. Essentially no information exists on differences between antiviral ribonucleoside metabolism in the cytoplasm relative to the mitochondrial matrix. However, entry of antiviral rNTPs into mitochondria through nucleotide transporters would eliminate an absolute requirement for intramitochondrial metabolism of antiviral ribonucleosides [23], [43]. To a first approximation, it is reasonable to assume that the total pool of antiviral rNTPs likely reflects the magnitude of the intramitochondrial pool as shown here for 2′-C-methyladenosine (**Table S1**).

The ability of TFIIS to excise most ribonucleotide analogues studied here demonstrates a robust, active mechanism in mammals to protect the integrity of RNA produced in the nucleus from insults by non-natural nucleotides (**Figure 3**). This property of TFIIS is even retained in yeast (**Figure S6**). Prokaryotes have similar proofreading factors that may serve a similar role. It is tempting to speculate that the evolution of these factors may have facilitated survival in environments where ribonucleoside analogues were used to secure a niche and fend off predator organisms.

The absence of an active mechanism for the removal of nucleotides with non-natural bases and ribose configurations and/or modifications incorporated by POLRMT suggests that it may be difficult to avoid off-target effects of antiviral ribonucleosides. The availability of biochemical tools to elucidate the nucleotide specificity of POLRMT empirically will contribute substantially to the identification of modifications capable of enhancing the specificity of antiviral ribonucleosides.

Manifestation of the side effects of NRTIs can take decades, perhaps a reflection of the substantial copy number of mtDNA, the quiescent nature of much mtDNA and/or the requirement for cell cycling to observe mtDNA replication [17], [22]–[24]. In contrast, expression of mtRNA is essential in all cells. Therefore, the manifestation of clinical signs in response to interference with mitochondrial transcription might be expected to occur more rapidly than observed for NRTIs. Interestingly, not all patients treated with the antiviral ribonucleosides under investigation here presented with adverse effects, suggesting that other conditions may contribute to a patient's response to antiviral ribonucleosides. One might imagine that individuals with undiagnosed mitochondrial disease would exhibit enhanced sensitivity to antiviral ribonucleotides capable of being incorporated into mitochondrial RNA. It is well documented that children with mitochondrial disease exhibit adverse effects to antibiotics like chloramphenicol for which the mitochondrial ribosome is an off target [44]. Evaluation of the exome and mtDNA sequences of patients exhibiting adverse effects to antiviral ribonucleosides may uncover susceptibility genes. If this hypothesis is correct, then antiviral ribonucleoside programs that have been discontinued may still have value for large segments of the population.

We propose the mitovir (*mito*chondrial dysfunction caused by anti*vir*al ribonucleosides) score as a parameter that can be determined easily during preclinical studies to identify antiviral ribonucleosides whose impact on mitochondrial function should be rigorously investigated (**Table 2**). This score is essentially an effective rate constant for utilization of the antiviral rNTP calculated by using the values for *k_pol_* and *K_d,app_* determined in vitro (**Table 2**) and the value for the concentration of antiviral rNTP present in cells (**Table 1**). The quotient of the mitovir score for the antiviral rNTP divided by that for the rNTP with which the antiviral rNTP competes yields an approximate frequency of incorporation of the antiviral ribonucleotide. Both of these parameters correlate well with values for CC_50_ measured in MT4 cells (**Figure 4C and 4D**). A correlation was more difficult to observe in Huh-7 cells (**Figure 4E**). The lack of robust reporting in Huh-7 cells may be a reflection that ATP levels are maintained by aerobic glycolysis [39]. It is possible that MT4 cells require mitochondrial function for growth in culture, even in the presence of high concentrations of glucose [39]. Because there is no net ATP produced in mammalian cells when galactose is used as the carbon source, oxidative phosphorylation becomes essential for cellular energetics [39]. Perhaps Huh-7 cells and other cell lines adapted for growth in galactose will be able to link antiviral ribonucleoside induced dysfunction of mitochondria to production of ATP and cell viability. Under these conditions, a stronger correlation with the mitovir score may be revealed. These findings also underscore the need to utilize cell lines in preclinical analogue screening studies that authentically reveal mitochondrial status.

The mitovir score for GTP or 2′-dATP, models for “misincorporation” events which POLRMT likely evolved to evade, indicate a frequency of misincorporation of 1 in 3,300 or 1 in 35,000, respectively (**Table 2**). Given that the primary coding transcript produced is on the order 16,000 nucleotides in length, the data suggest that transition mutations are better tolerated than altered ribose configuration. Lethal mutagens like ribavirin may therefore represent a class of antiviral agents with a higher cellular tolerability. The mitovir score for ribavirin predicts a frequency of incorporation of 1 in 160,000, perhaps establishing an upper limit for an antiviral ribonucleoside that is miscoding but fails to inhibit transcription elongation.

The case of 4′-azidocytidine supports the utility of the mitovir score. Preclinical studies, including long-term studies in mice and dogs, failed to reveal any toxicity [4], [10]. Indeed, even in MT4 cells, the value for the CC_50_ was high corresponding to the low intracellular level of triphosphorylated metabolite (**Table 1**). The mitovir score predicts an incorporation frequency of 1 in 700 nucleotides (**Table 2**). This value suggests a clear, negative impact on mitochondrial transcription in cells (**Figure 2D**). While the mitovir scores are not as high as those for 4′-azidocytidine, we've found that 2′-C-methyl analogues are substrates for POLRMT and that 2′-C-methyladenosine-triphosphate is formed within mitochondria and selectively inhibits production of mitochondrial transcripts in cells. The relevance of these findings to clinical observation with related compounds requires further study. In contrast, the currently used nucleoside analog ribavirin was found to be an exceedingly poor substrate for POLRMT. We have also found 2′-deoxy-2′-fluoro-2′-C-methyluridine-triphosphate, formed by mericitabine [45] and GS-7977 [34], to be an even worse substrate for POLRMT than ribavirin triphosphate. The combined biochemical and biological approaches reported here should facilitate the development of highly efficacious therapeutic ribonucleotide analogues with lower potential for toxicity.

## Materials and Methods

### Human mitochondrial RNA polymerase nucleotide incorporation assays

Nucleotide incorporation experiments were performed as described previously [30]. Briefly, elongation complexes were formed by incubating POLRMT with the appropriate primed template, mixed with appropriate nucleoside triphosphate substrate and quenched by addition of EDTA. Products were analyzed be denaturing PAGE and quantitated by phosphorimaging. All details and equations employed are provided in **Protocol S1**. POLRMT was expressed and purified as described previously [30]. POLRMT of comparable activity and purity can be obtained from INDIGO Biosciences, Inc. (State College, PA).

### Pol II elongation complex assembly and transcription elongation assays

The elongation complexes of yeast or bovine Pol II were assembled with RNA and DNA oligonucleotides as indicated in the Figure Legends. The assembly and purification of the elongation complexes was performed by membrane filtration as previously described in detail [37]. Transcription elongation assays and the detection of transcription products were performed as described previously [37], [38]. Human recombinant TFIIS was expressed and purified as described in [46]. Yeast TFIIS was expressed and purified according to [47] using expression construct generously provided by C. Kane.

### HCV NS5B enzymatic assays

HCV NS5B-dependent RNA elongation activity was assayed using a heteropolymeric RNA template as described previously [48]. Detailed protocols are provided in **Protocol S1**.

### Cell lines

Huh-7 cells were maintained in growth medium containing Dulbecco's Modified Eagle Medium (DMEM) with GlutaMAX (Gibco, Carlsbad, CA), supplemented with 10% fetal bovine serum (FBS) (HyClone, Logan, UT), 100 units/mL penicillin, 100 µg/mL streptomycin (Gibco, Carlsbad, CA) and 0.1 mM non-essential amino acids (Gibco, Carlsbad, CA). For Huh-7 cells grown in galactose media, growth medium contained DMEM (-glucose) (Gibco, Carlsbad, CA), supplemented with 5 mM HEPES (Sigma), 10 mM galactose (Sigma), 1 mM sodium pyruvate (Cellgro), 10% FBS, 100 units/mL penicillin and 100 µg/mL streptomycin. Cells were adapted for 3 weeks in galactose media before treating with compounds. MT-4 cells were obtained from the NIH AIDS Research and Reference Reagent Program (Germantown, MD), and maintained in RPMI-1640 medium (IrvineScientific, Santa Ana, CA) supplemented with 100 units/mL penicillin, 100 µg/mL streptomycin, 2 nM L-glutamine and 10% FBS. MT-4 cells were passed twice a week and maintained at densities below 0.6×10^6^ cells/mL. HeLa S3 cells were obtained from ATCC (Manassas, VA), and maintained in DMEM supplemented with 10% FBS, 100 units/mL penicillin and 100 µg/mL streptomycin.

### Intracellular metabolism

Cells were treated for 24 h with 10 µM of each ribonucleoside analog. For analogs that caused toxicity at 10 µM, triphosphate levels had to be dose normalized after being determined with incubations at 0.1 µM. After 24 hours, cells were counted and isolated by spinning through oil essentially as previously described [49], except that the oil layer was washed with ice cold normal saline instead of phosphate buffered saline. After storing samples in 70% methanol at −20°C for at least overnight to facilitate cell lysis, cell (Huh-7 and MT4) or mitochondrial (from HeLa S3 cells) lysates were dried and suspended in 3 mM ammonium formate (pH 5) with 10 mM dimethylhexylamine in water at a concentration of extract from 100,000 cells/10 µL for Huh-7 and MT4 cells or extract from mitochondria isolated from 1 million cells/10 µL for mitochondria isolated from HeLa S3 cells and 10 µL of the samples were analyzed by LC/MS/MS. Ribonucleoside triphosphates were quantified by ion pairing LC coupled to positive mode MS/MS essentially as described previously [50]. Detailed protocols are provided in **Protocol S2**.

### Cytotoxicity assays

The 5-day cytotoxicity of compounds was performed using CellTiter Glo viability reagents (Promega). Detailed protocols are provided in **Protocol S2**.

### HCV replicon assays

A Huh-7 cell line carrying a Renilla luciferase HCV genotype 1b replicon was used to assess antiviral activity of compounds as described previously [51], [52]. Detailed protocols are provided in **Protocol S2**.

### Mitochondrial transcription in cells

Cells were treated with 50 ng/mL ethidium bromide and placed at 37°C in a 5% CO_2_ incubator to deplete mitochondrial transcripts from cells. After 24 h, media was removed, cells were washed with PBS and fresh media was added containing the appropriate compound at various concentrations. DMSO at 0.5% was used as a negative control. Cells were placed at 37°C in a 5% CO_2_ incubator for one, two or three days. Every 24 h the media was removed and replaced with fresh media containing the appropriate compound. At the indicated time point, media was removed, cells washed with PBS, harvested and Northern blots performed.

### Northern blot analysis

Northern blots were performed for the detection of mitochondrial transcripts ND1 and ND5 and nuclear transcript GAPDH. Cells were lysed by adding TRI Reagent (Sigma, Cat# T94247), RNA isolated and separated on a 1% denaturing agarose gel. RNA was transferred to nylon membrane, slightly dried and RNA was crosslinked to the membrane using a UV Crosslinker (Stratalinker 2400, Stratagene). Hybridization was performed in a modified Church's buffer for 16 h at 65°C. Membranes were visualized by using a PhosphorImager (GE). Hybridization probes were made by PCR. Detailed protocols are provided in **Protocol S2**.

### Statistical analysis

Statistical analyses were performed using GraphPad Prism 5.0 (La Jolla, CA). Nonparametric Spearman correlation coefficients (r) were computed and from these values one-tailed tests for statistical significance (P-values) were calculated. The s.d. and s.e.m values are indicated where appropriate.

## Supporting Information

Figure S1 **Kinetic parameters for POLRMT-catalyzed nucleotide incorporation: Adenosine analogs.** Kinetics of nucleotide incorporation as a function of nucleoside triphosphate concentration for (**A**) ATP, (**B**) 2′-C-methyl-ATP, (**C**) 7-deaza-ATP, (**D**) 3-deaza-ATP, (**E**) 3′-dATP, (**F**) 6-methylpurine-TP and (**G**) ribavirin-TP. Observed rate constants (*k*_obs_) for nucleotidyl transfer at various concentrations of nucleotide substrate were obtained by fitting either product-versus-time data or relative fluorescence-versus-time data to an equation defining a single exponential. Values for k_obs_ were then plotted as a function of nucleoside triphosphate concentration and fit to a hyperbola, yielding the maximal rate constant for incorporation (*k_pol_*) and apparent dissociation constant (*K_d,app_*).
(PDF)

Figure S2 **Kinetic parameters for POLRMT-catalyzed nucleotide incorporation: Cytidine analogs.** Kinetics of nucleotide incorporation as a function of nucleoside triphosphate concentration for (**A**) CTP, (**B**) 2′-C-methyl-CTP, (**C**) 2′-deoxy-2′-fluoro-CTP, (**D**) 4′-methyl-CTP and (**E**) 4′-azido-CTP. Observed rate constants (*k*_obs_) for nucleotidyl transfer at various concentrations of nucleotide substrate were obtained by fitting either product-versus-time data or relative fluorescence-versus-time data to an equation defining a single exponential. Values for k_obs_ were then plotted as a function of nucleoside triphosphate concentration and fit to a hyperbola, yielding the maximal rate constant for incorporation (*k_pol_*) and apparent dissociation constant (*K_d,app_*).
(PDF)

Figure S3 **Kinetic parameters for POLRMT-catalyzed nucleotide incorporation: Guanine and uridine analogs.** Kinetics of nucleotide incorporation as a function of nucleoside triphosphate concentration for (**A**) GTP, (**B**) 2′-C-methyl-GTP, (**C**) UTP and (**D**) 2′-deoxy-2′-fluoro-2′-C-methyl-UTP. Observed rate constants (*k*_obs_) for nucleotidyl transfer at various concentrations of nucleotide substrate were obtained by fitting either product-versus-time data or relative fluorescence-versus-time data to an equation defining a single exponential. Values for k_obs_ were then plotted as a function of nucleoside triphosphate concentration and fit to a hyperbola, yielding the maximal rate constant for incorporation (*k_pol_*) and apparent dissociation constant (*K_d,app_*).
(PDF)

Figure S4 **Production of full-length mitochondrial RNA transcripts is impaired in the presence of 2′-C-methyladenosine: Overexposed Northern blots showing a ladder of truncated products.** Northern blot of ND5 after EtBr treatment and recovery in the presence of 2′-C-methyladenosine. The blot was overexposed and shows a ladder of truncated RNA products that accumulate over time from cells treated with 2′-C-methyladenosine.
(PDF)

Figure S5 **Kinetics of TFIIS-mediated RNA cleavage.** (**A,B**) Kinetics of TFIIS-mediated RNA cleavage subsequent to AMP, 2′C-methyl-AMP and 3′-dAMP incorporation by calf thymus Pol II. TEC-C12 was incubated with 500 µM ATP, 2′-C-methyl-ATP or 3′-dATP for 2 min at which point TFIIS added to 1 µM final concentration for the indicated time. The percentage of A13 remaining was plotted as a function of time and fit to a single exponential yielding observed rate constants of TFIIS-mediated RNA cleavage of 0.040±0.006, 0.0060±0.0020, 0.14±0.02 s^−1^ after incorporation of AMP, 2′-C-methyl-AMP or 3′-dAMP respectively. (**C,D**) Kinetics of TFIIS-mediated RNA cleavage subsequent to CMP, 2′C-methyl-CMP and 4′-azido-CMP incorporation. TEC-A11 was incubated with 500 µM CTP or 2′-C-methyl-CTP for 2 min or 4′-azido-CTP for 10 min at which point TFIIS added to 1 or 10 µM final concentration for the indicated time. The percentage of C12 remaining was plotted as a function of time and fit to a single exponential yielding observed rate constants of TFIIS-mediated RNA cleavage of 0.088±0.003, 0.0018±0.0003, 0.13±0.01 and 0.13±0.01 s^−1^ after incorporation of CMP, 2′-C-methyl-CMP or4′-azido-CMP respectively. At 10 µM TFIIS the observed rate constant of TFIIS-mediated RNA cleavage after incorporation of 2′-C-methyl-CMP was 0.0045±0.0004 s^−1^.
(PDF)

Figure S6 **Nucleoside analog incorporation and extension of the resulting products catalyzed by yeast Pol II.** (**A**) Schematic of synthetic nucleic scaffolds for transcription elongation complex (TECs) assembly with yeast Pol II. The TECs were assembled using TDS76 and NDS79 and either RNA9 in the presence of 10 µM GTP (TEC-G10) or RNA 7(20) in the presence of 10 µM each ATP, GTP and UTP (TEC-A11); the TECs were purified from the unincorporated DNA, RNA and NTPs before addition of the appropriate nucleoside analog triphosphate. The oligonucleotide sequences are in **Table S2**. (**B,C**) Reaction products from Pol II-catalyzed nucleotide incorporation in the absence and presence of TFIIS. The concentration of the unmodified substrate NTP and analogs were 500 µM; TFIIS was added at 10 µM. Reactions proceeded for 1 min. Reaction with ribavirin-TP proceeded for 10 min. (**D,E**) Reaction products from Pol II-catalyzed nucleotide incorporation in the presence of the next correct nucleotide substrate. The concentration of the unmodified substrate NTP and analogs were 500 µM; TFIIS was added at 10 µM. Reactions proceeded for 1 min. Reaction with ribavirin-TP proceeded for 10 min. (**F**) Percent inhibition by TFIIS on Pol II nucleoside analog incorporation.
(PDF)

Protocol S1 **Polymerase-catalyzed nucleotide incorporation assays.**
(DOCX)

Protocol S2 **Evaluation of nucleoside analogs in cells.**
(DOCX)

Table S1 **Mitochondrial ribonucleoside triphosphate concentrations.**
(DOCX)

Table S2 **Oligonucleotides used for the elongation complex assembly.**
(DOCX)
