# Supplementary material for: Sensitivity of Mitochondrial Transcription and Resistance of RNA Polymerase II Dependent Nuclear Transcription to Antiviral Ribonucleosides
Source: PLoS Pathog. 2012 Nov 15;8(11):e1003030. doi: 10.1371/journal.ppat.1003030 (PMC3499576; doi:10.1371/journal.ppat.1003030)
Supplement: Figure S1 — Kinetic parameters for POLRMT-catalyzed nucleotide incorporation: Adenosine analogs. Kinetics of nucleotide incorporation as a function of nucleoside triphosphate concentration for (A) ATP, (B) 2′-C-methyl-ATP, (C) 7-deaza-ATP, (D) 3-deaza-ATP, (E) 3′-dATP, (F) 6-methylpurine-TP and (G) ribavirin-TP. Observed rate constants (k obs) for nucleotidyl transfer at various concentrations of nucleotide substrate were obtained by fitting either product-versus-time data or relative fluorescence-versus-time data to an equation defining a single exponential. Values for kobs were then plotted as a function of nucleoside triphosphate concentration and fit to a hyperbola, yielding the maximal rate constant for incorporation (kpol) and apparent dissociation constant (Kd,app). (PDF) [file ppat.1003030.s001.pdf]

Fig. S1

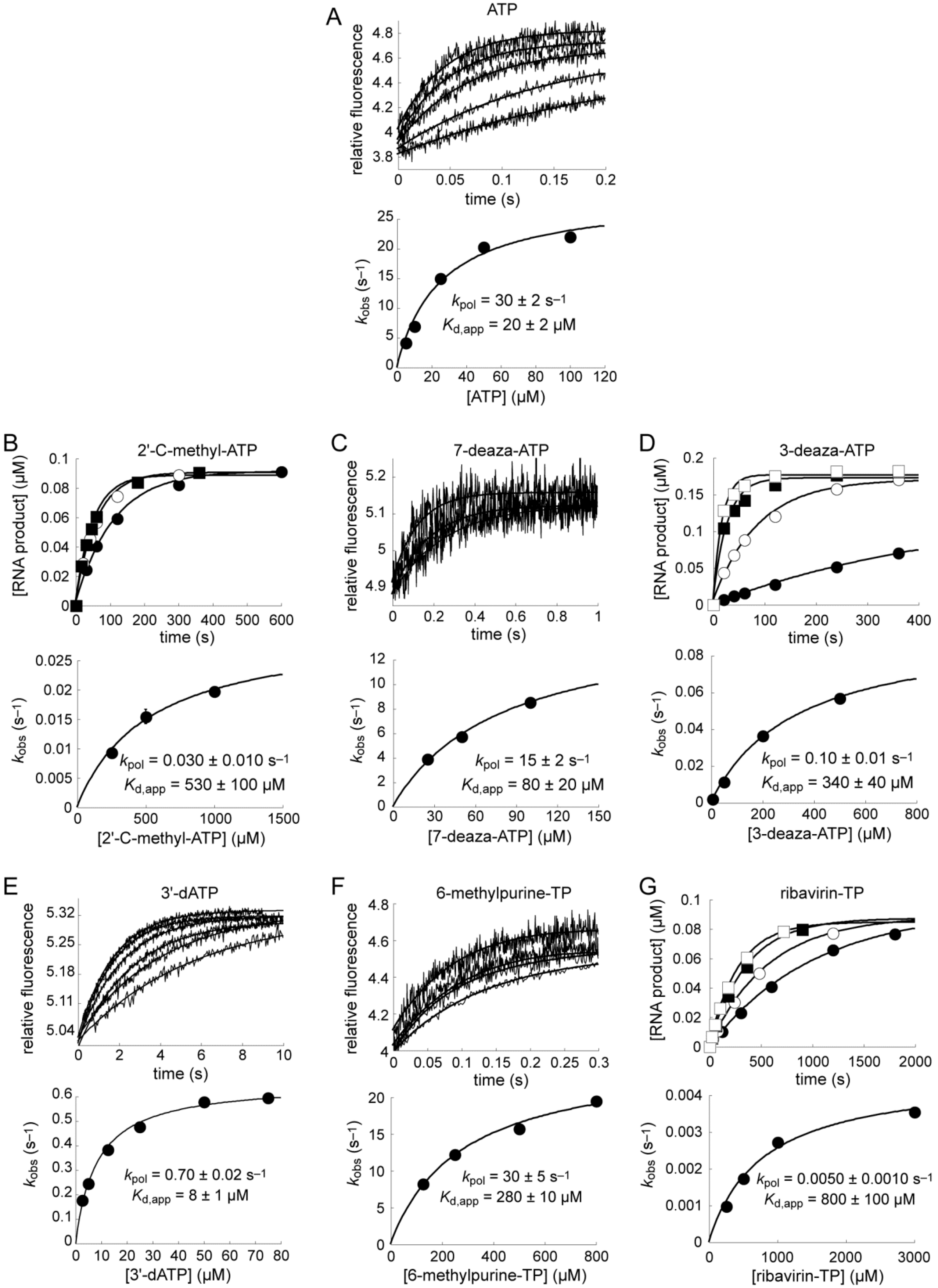
A
ATP
relative fluorescence
time (s)
k_{obs} (s–1)
k_{pol} = 30 ± 2 s–1
$K_{d,app}$ = 20 ± 2 μM
[ATP] (μM)
B
2'-C-methyl-ATP
[RNA product] (μM)
time (s)
k_{obs} (s–1)
k_{pol} = 0.030 ± 0.010 s–1
$K_{d,app}$ = 530 ± 100 μM
[2'-C-methyl-ATP] (μM)
C
7-deaza-ATP
relative fluorescence
time (s)
k_{obs} (s–1)
k_{pol} = 15 ± 2 s–1
$K_{d,app}$ = 80 ± 20 μM
[7-deaza-ATP] (μM)
D
3-deaza-ATP
[RNA product] (μM)
time (s)
k_{obs} (s–1)
k_{pol} = 0.10 ± 0.01 s–1
$K_{d,app}$ = 340 ± 40 μM
[3-deaza-ATP] (μM)
E
3'-dATP
relative fluorescence
time (s)
k_{obs} (s–1)
k_{pol} = 0.70 ± 0.02 s–1
$K_{d,app}$ = 8 ± 1 μM
[3'-dATP] (μM)
F
6-methylpurine-TP
relative fluorescence
time (s)
k_{obs} (s–1)
k_{pol} = 30 ± 5 s–1
$K_{d,app}$ = 280 ± 10 μM
[6-methylpurine-TP] (μM)
G
ribavirin-TP
[RNA product] (μM)
time (s)
k_{obs} (s–1)
k_{pol} = 0.0050 ± 0.0010 s–1
$K_{d,app}$ = 800 ± 100 μM
[ribavirin-TP] (μM)

Figure S1. Kinetic Parameters for POLRMT-Catalyzed Nucleotide Incorporation: Adenosine Analogs. (**A**) Correct AMP incorporation. POLRMT (0.125 µM) was incubated with 8 bp 2AP scaffold (0.1 µM) for 3 min and then rapidly mixed with ATP (5, 10, 25, 50 or 100 µM) using a stopped-flow. The observed change in fluorescence emission was measured and fit to a single exponential (Eq. 1), yielding k_{obs} values of 4 ± 1, 7 ± 1, 15 ± 1, 20 ± 1 and 22 ± 1 s^{-1} for 5, 10, 25, 50 or 100 µM ATP, respectively. Values for k_{obs} were plotted as a function of ATP concentration and fit to a hyperbola (Eq. 2), yielding a k_{pol} value of 30 ± 1 s^{-1} and a $K_{d,app}$ value of 20 ± 2 µM. (**B**) 2′-C-methyl-AMP misincorporation. POLRMT (0.5 µM) was incubated with 5′-^{32}P-labeled-RNA/DNA 8 bp scaffold (0.1 µM) for 3 min and then rapidly mixed with 2′-C-methyl-ATP (250, 500 or 1000 µM). Reactions were quenched at various times with EDTA (300 mM). Quantitated RNA product was plotted as a function of time and fit to a single exponential (Eq. 1) yielding values for k_{obs} of 0.0093 ± 0.0006, 0.015 ± 0.001 and 0.019 ± 0.001 s^{-1} for 250 (●), 500 (○) or 1000 (■) µM 2′-C-methyl-ATP, respectively. Values for k_{obs} were plotted as a function of 2′-C-methyl-ATP concentration and fit to a hyperbola (Eq. 2), yielding a k_{pol} value of 0.030 ± 0.010 s^{-1} and a $K_{d,app}$ value of 530 ± 100 µM. (**C**) 7-deaza-AMP misincorporation. POLRMT (0.25 µM) was incubated with 8 bp 2AP scaffold (0.125 µM) for 3 min and then rapidly mixed with 7-deaza-ATP (25, 50, or 100 µM) using a stopped-flow. The observed change in fluorescence emission was measured and fit to a single exponential (Eq. 1), yielding k_{obs} values of 3.91 ± 0.22, 5.56 ± 0.26, and 8.1 ± 0.5 s^{-1} for 25, 50, or 100 µM 7-deaza-ATP, respectively. Values for k_{obs} were plotted as a function of 7-deaza-ATP concentration and fit to a hyperbola (Eq. 2), yielding a k_{pol} value of 15 ± 2 s^{-1} and a $K_{d,app}$ value of 80 ± 20 µM. (**D**) 3-deaza-AMP misincorporation. POLRMT (0.5 µM) was incubated with 5′-^{32}P-labeled-RNA/DNA 8 bp scaffold (0.1 µM) for 3 min and then rapidly mixed with 3-deaza-ATP (5, 50, 200 or 500 µM). Reactions were quenched at various times with EDTA (300 mM). Quantitated RNA product was plotted as a function of time and fit to a single exponential (Eq. 1) yielding values for k_{obs} of 0.0020 ± 0.0004, 0.011 ± 0.001, 0.036 ± 0.006 and 0.057 ± 0.007 s^{-1} for 5 (●), 50 (○), 200 (■) or 500 (□) µM 3-deaza-ATP, respectively. Values for k_{obs} were plotted as a function of 3-deaza-ATP concentration and fit to a hyperbola (Eq. 2), yielding a k_{pol} value of 0.10 ± 0.01 s^{-1} and a $K_{d,app}$ value of 340 ± 40 µM. (**E**) 3′-dAMP misincorporation. POLRMT (0.25 µM) was incubated with 8 bp 2AP scaffold (0.125 µM) for 3 min and then rapidly mixed with 3′-dATP (2.5, 5, 12.5, 25, 50 or 75 µM) plus 1.25 µM single-strand DNA trap using a stopped-flow apparatus. The observed change in fluorescence emission was measured and fit to a single exponential (Eq. 1), yielding k_{obs} values of 0.18 ± 0.01, 0.25 ± 0.02, 0.38 ± 0.03, 0.48 ± 0.03, 0.58 ± 0.04 and 0.60 ± 0.04 s^{-1} for 2.5, 5, 12.5, 25, 50 or 75 µM 3′-dATP, respectively. Values for k_{obs} were plotted as a function of 3′-dATP concentration and fit to a hyperbola (Eq. 2), yielding a kpol of 0.70 ± 0.02 s^{-1} and a $K_{d,app}$ value of 8 ± 1 µM. (**F**) 6-methylpurine misincorporation. POLRMT (0.25 µM) was incubated with 8 bp 2AP scaffold (0.125 µM) for 3 min and then rapidly mixed with 6-methylpurine-TP (100, 250, 500 or 800 µM) using a stopped-flow. The observed change in fluorescence emission was measured and fit to a single exponential (Eq. 1), yielding k_{obs} values of 8.2 ± 0.2, 12.2 ± 0.5, 15.7 ± 0.5 and 19.5 ± 0.5 s^{-1} for 100, 250, 500 or 800 µM 6-methylpurine-TP, respectively. Values for k_{obs} were plotted as a function of 6-methylpurine-TP concentration and fit to a hyperbola (Eq. 2), yielding a k_{pol} value of 30 ± 5 s^{-1} and a $K_{d,app}$ value of 280 ± 10 µM. (**G**) ribavirin misincorporation. POLRMT (0.5 µM) was incubated with 5′-^{32}P-labeled-RNA/DNA 8 bp scaffold (0.1 µM) for 3 min and then rapidly mixed with ribavirin-TP (250, 500, 1000 or 3000 µM). Reactions were quenched at various times with EDTA (300 mM). Quantitated RNA product was plotted as a function of time and fit to a single exponential (Eq. 1) yielding values for k_{obs} of 0.00098 ± 0.00006, 0.0017 ± 0.0004, 0.0027 ± 0.0001 and 0.0036 ± 0.0001 s^{-1} for 250 (●), 500 (○), 1000 (■) or 3000 (□) µM ribavirin-TP, respectively. Values for k_{obs} were plotted as a function of ribavirin-TP concentration and fit to a hyperbola (Eq. 2), yielding a k_{pol} value of 0.0050 ± 0.0010 s^{-1} and a $K_{d,app}$ value of 800 ± 100 µM.
