# Supplementary material for: Sensitivity of Mitochondrial Transcription and Resistance of RNA Polymerase II Dependent Nuclear Transcription to Antiviral Ribonucleosides
Source: PLoS Pathog. 2012 Nov 15;8(11):e1003030. doi: 10.1371/journal.ppat.1003030 (PMC3499576; doi:10.1371/journal.ppat.1003030)
Supplement: Figure S2 — Kinetic parameters for POLRMT-catalyzed nucleotide incorporation: Cytidine analogs. Kinetics of nucleotide incorporation as a function of nucleoside triphosphate concentration for (A) CTP, (B) 2′-C-methyl-CTP, (C) 2′-deoxy-2′-fluoro-CTP, (D) 4′-methyl-CTP and (E) 4′-azido-CTP. Observed rate constants (k obs) for nucleotidyl transfer at various concentrations of nucleotide substrate were obtained by fitting either product-versus-time data or relative fluorescence-versus-time data to an equation defining a single exponential. Values for kobs were then plotted as a function of nucleoside triphosphate concentration and fit to a hyperbola, yielding the maximal rate constant for incorporation (kpol) and apparent dissociation constant (Kd,app). (PDF) [file ppat.1003030.s002.pdf]

Fig. S2

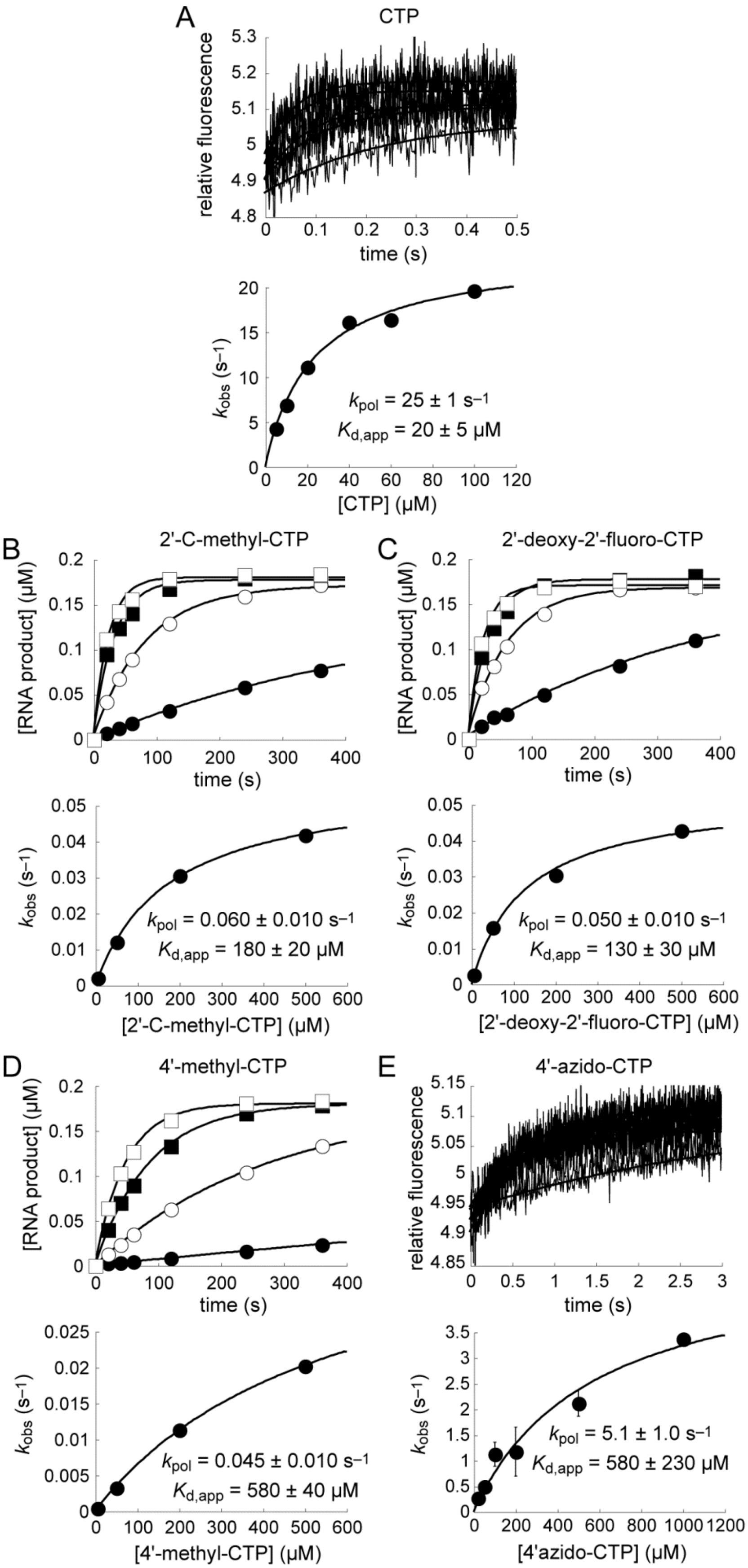
A
CTP
relative fluorescence
time (s)
k_{obs} (s−1)
k_{pol} = 25 ± 1 s−1
$K_{d,app}$ = 20 ± 5 μM
[CTP] (μM)
B
2'-C-methyl-CTP
[RNA product] (μM)
time (s)
k_{obs} (s−1)
k_{pol} = 0.060 ± 0.010 s−1
$K_{d,app}$ = 180 ± 20 μM
[2'-C-methyl-CTP] (μM)
C
2'-deoxy-2'-fluoro-CTP
[RNA product] (μM)
time (s)
k_{obs} (s−1)
k_{pol} = 0.050 ± 0.010 s−1
$K_{d,app}$ = 130 ± 30 μM
[2'-deoxy-2'-fluoro-CTP] (μM)
D
4'-methyl-CTP
[RNA product] (μM)
time (s)
k_{obs} (s−1)
k_{pol} = 0.045 ± 0.010 s−1
$K_{d,app}$ = 580 ± 40 μM
[4'-methyl-CTP] (μM)
E
4'-azido-CTP
relative fluorescence
time (s)
k_{obs} (s−1)
k_{pol} = 5.1 ± 1.0 s−1
$K_{d,app}$ = 580 ± 230 μM
[4'azido-CTP] (μM)

Figure S2. Kinetic Parameters for POLRMT-Catalyzed Nucleotide Incorporation: Cytidine Analogs. (**A**) Correct CMP incorporation. POLRMT (0.125 μM) was incubated with 8 bp 2AP scaffold (0.1 μM) for 3 min and then rapidly mixed with CTP (5, 10, 25, 50 or 100 μM) using a stopped-flow. The observed change in fluorescence emission was measured and fit to a single exponential (Eq. 1), yielding k_{obs} values of 4.3 ± 0.3, 9.0 ± 0.9, 11.5 ± 0.9, 17.3 ± 1.5 and 17.8 ± 2.2 s^{-1} for 5, 10, 20, 40 or 100 μM CTP, respectively. Values for k_{obs} were plotted as a function of CTP concentration and fit to a hyperbola (Eq. 2), yielding a k_{pol} value of 25 ± 1 s^{-1} and a $K_{d,app}$ value of 20 ± 5 μM. (**B**) 2′-C-methyl-CMP misincorporation. POLRMT (0.5 μM) was incubated with 5′-^{32}P-labeled-RNA/DNA 8 bp scaffold (0.1 μM) for 3 min and then rapidly mixed with 2′-C-methyl-CTP (5, 50, 200 or 500 μM). Reactions were quenched at various times with EDTA (300 mM). Quantitated RNA product was plotted as a function of time and fit to a single exponential (Eq. 1) yielding values for k_{obs} of 0.0021 ± 0.0002, 0.012 ± 0.0007, 0.031 ± 0.004 and 0.042 ± 0.004 s^{-1} for 5 (●), 50 (○) 200 (■) or 500 (□) μM 2′-C-methyl-CTP, respectively. Values for k_{obs} were plotted as a function of 2′-C-methyl-CTP concentration and fit to a hyperbola (Eq. 2), yielding a k_{pol} value of 0.060 ± 0.010 s^{-1} and a $K_{d,app}$ value of 180 ± 20 μM. (**C**) 2′-deoxy-2′-fluoro-CMP misincorporation. POLRMT (0.5 μM) was incubated with 5′-^{32}P-labeled-RNA/DNA 8 bp scaffold (0.1 μM) for 3 min and then rapidly mixed with 2′-deoxy-2′-fluoro-CTP (5, 50, 200 or 500 μM). Reactions were quenched at various times with EDTA (300 mM). Quantitated RNA product was plotted as a function of time and fit to a single exponential (Eq. 1) yielding values for k_{obs} of 0.0026 ± 0.0007, 0.016 ± 0.002, 0.030 ± 0.003 and 0.043 ± 0.004 s^{-1} for 5 (●), 50 (○) 200 (■) or 500 (□) μM 2′-deoxy-2′-fluoro-CTP, respectively. Values for k_{obs} were plotted as a function of 2′-deoxy-2′-fluoro-CTP concentration and fit to a hyperbola (Eq. 2), yielding a k_{pol} value of 0.050 ± 0.010 s^{-1} and a $K_{d,app}$ value of 130 ± 30 μM. (**D**) 4′-methyl-CMP misincorporation. POLRMT (0.5 μM) was incubated with 5′-^{32}P-labeled-RNA/DNA 8 bp scaffold (0.1 μM) for 3 min and then rapidly mixed with 4′-methyl-CTP (5, 50, 200 or 500 μM). Reactions were quenched at various times with EDTA (300 mM). Quantitated RNA product was plotted as a function of time and fit to a single exponential (Eq. 1) yielding values for k_{obs} of 0.00040 ± 0.00007, 0.0036 ± 0.0003, 0.012 ± 0.002 and 0.021 ± 0.004 s^{-1} for 5 (●), 50 (○) 200 (■) or 500 (□) μM 4′-methyl-CTP, respectively. Values for k_{obs} were plotted as a function of 4′-methyl-CTP concentration and fit to a hyperbola (Eq. 2), yielding a k_{pol} value of 0.045 ± 0.010 s^{-1} and a $K_{d,app}$ value of 580 ± 40 μM. (**E**) 4′-azido-CMP misincorporation. POLRMT (0.125 μM) was incubated with 8 bp 2AP scaffold (0.1 μM) for 3 min and then rapidly mixed with 4′-azido-CTP (20, 50, 100, 200 or 500 μM) using a stopped-flow. The observed change in fluorescence emission was measured and fit to a single exponential (Eq. 1), yielding k_{obs} values of 0.27 ± 0.01, 0.50 ± 0.06, 1.13 ± 0.02, 1.12 ± 0.05 and 2.12 ± 0.25 s^{-1} for 20, 50, 100, 200 or 500 μM 4′-azido-CTP, respectively. Values for k_{obs} were plotted as a function of 4′-azido-CTP concentration and fit to a hyperbola (Eq. 2), yielding a k_{pol} value of 5.1 ± 1.0 s^{-1} and a $K_{d,app}$ value of 580 ± 230 μM.
