# Supplementary material for: Sensitivity of Mitochondrial Transcription and Resistance of RNA Polymerase II Dependent Nuclear Transcription to Antiviral Ribonucleosides
Source: PLoS Pathog. 2012 Nov 15;8(11):e1003030. doi: 10.1371/journal.ppat.1003030 (PMC3499576; doi:10.1371/journal.ppat.1003030)
Supplement: Figure S3 — Kinetic parameters for POLRMT-catalyzed nucleotide incorporation: Guanine and uridine analogs. Kinetics of nucleotide incorporation as a function of nucleoside triphosphate concentration for (A) GTP, (B) 2′-C-methyl-GTP, (C) UTP and (D) 2′-deoxy-2′-fluoro-2′-C-methyl-UTP. Observed rate constants (k obs) for nucleotidyl transfer at various concentrations of nucleotide substrate were obtained by fitting either product-versus-time data or relative fluorescence-versus-time data to an equation defining a single exponential. Values for kobs were then plotted as a function of nucleoside triphosphate concentration and fit to a hyperbola, yielding the maximal rate constant for incorporation (kpol) and apparent dissociation constant (Kd,app). (PDF) [file ppat.1003030.s003.pdf]

Fig. S3

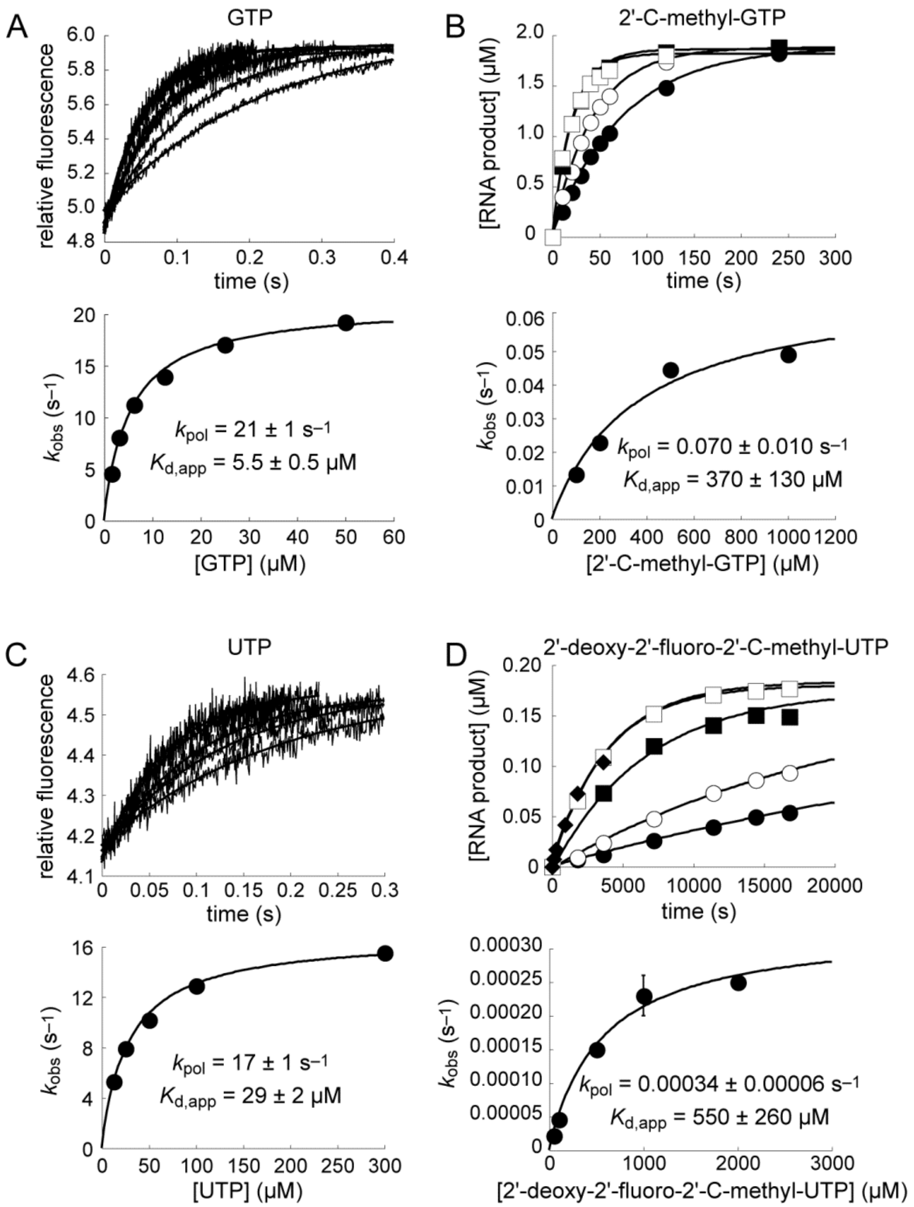

Figure S3. Kinetic Parameters for POLRMT-Catalyzed Nucleotide Incorporation: Guanine and Uridine Analogs. (**A**) Correct GMP incorporation. POLRMT (0.125 μM) was incubated with 8 bp 2AP scaffold (0.1 μM) for 3 min and then rapidly mixed with GTP (1.56, 3.12, 6.25, 12.5, 25 or 50 μM) using a stopped-flow. The observed change in fluorescence emission was measured and fit to a single exponential (Eq. 1), yielding k_{obs} values of 4.6 ± 0.1, 8.0 ± 0.1, 11.5 ± 0.1, 14.0 ± 0.2, 17.1 ± 0.2 and 19.0 ± 0.2 s^{-1} for 1.56, 3.12, 6.25, 12.5, 25 or 50 μM GTP, respectively. Values for k_{obs} were plotted as a function of GTP concentration and fit to a hyperbola (Eq. 2), yielding a k_{pol} value of 21 ± 1 s^{-1} and a $K_{d,app}$ value of 5.5 ± 0.5 μM. (**B**) 2′-C-methyl-GMP misincorporation. POLRMT (3.6 μM) was incubated with 5′-^{32}P-labeled-RNA/DNA 8 bp scaffold (2 μM) for 3 min and then rapidly mixed with 2′-C-methyl-GTP (100, 200, 500 or 1000 μM). Reactions were quenched at various times with EDTA (300 mM). Quantitated RNA product was plotted as a function of time and fit to a single exponential (Eq. 1) yielding values for k_{obs} of 0.013 ± 0.001, 0.023 ± 0.001, 0.045 ± 0.002 and 0.049 ± 0.005 s^{-1} for 100 (●), 200 (○) 500 (■) or 1000 (□) μM 2′-C-methyl-GTP, respectively. Values for k_{obs} were plotted as a function of 2′-C-methyl-CTP concentration and fit to a hyperbola (Eq. 2), yielding a k_{pol} value of 0.070 ± 0.010 s^{-1} and a $K_{d,app}$ value of 370 ± 130 μM. (**C**) Correct UMP incorporation. POLRMT (0.125 μM) was incubated with 8 bp 2AP scaffold (0.1 μM) for 3 min and then rapidly mixed with UTP (12.5, 25, 50, 100 or 300

μM) using a stopped-flow. The observed change in fluorescence emission was measured and fit to a single exponential (Eq. 1), yielding k_{obs} values of 5.3 ± 0.2, 7.9 ± 0.2, 10.2 ± 0.3, 12.9 ± 0.4 and 15.5 ± 0.5 s^{-1} for 12.5, 25, 50, 100 or 300 μM UTP, respectively. Values for k_{obs} were plotted as a function of UTP concentration and fit to a hyperbola (Eq. 2), yielding a k_{pol} value of 17 ± 1 s^{-1} and a $K_{d,app}$ value of 29 ± 2 μM. (**D**) 2′-deoxy-2′-fluoro-2′-C-methyl-UMP misincorporation. POLRMT (0.5μM) was incubated with 5′-^{32}P-labeled-RNA/DNA 8 bp scaffold (0.2 μM) for 3 min and then rapidly mixed with 2′-deoxy-2′-fluoro-2′-C-methyl-UTP (100, 200, 500, 1000 or 2000 μM). Reactions were quenched at various times with EDTA (300 mM). Quantitated RNA product was plotted as a function of time and fit to a single exponential (Eq. 1) yielding values for k_{obs} of 2.2 ± 0.2 x 10^{-5}, 4.6 ± 0.2 x 10^{-5}, 1.5 ± 0.2 x 10^{-4}, 2.6 ± 0.3 x $10^{-4}$and 2.5 ± 0.2 x 10^{-4} s^{-1} for 100 (●), 200 (○) 500 (■), 1000 (□) or 2000 (◆) μM 2′-deoxy-2′-fluoro-2′-C-methyl-UTP, respectively. Values for k_{obs} were plotted as a function of 2′-deoxy-2′-fluoro-2′-C-methyl-UTP concentration and fit to a hyperbola (Eq. 2), yielding a k_{pol} value of 3.4 ± 0.6 x 10^{-4} s^{-1} and a $K_{d,app}$ value of 550 ± 260 μM.
