# Supplementary material for: Sensitivity of Mitochondrial Transcription and Resistance of RNA Polymerase II Dependent Nuclear Transcription to Antiviral Ribonucleosides
Source: PLoS Pathog. 2012 Nov 15;8(11):e1003030. doi: 10.1371/journal.ppat.1003030 (PMC3499576; doi:10.1371/journal.ppat.1003030)
Supplement: Figure S4 — Production of full-length mitochondrial RNA transcripts is impaired in the presence of 2′-C-methyladenosine: Overexposed Northern blots showing a ladder of truncated products. Northern blot of ND5 after EtBr treatment and recovery in the presence of 2′-C-methyladenosine. The blot was overexposed and shows a ladder of truncated RNA products that accumulate over time from cells treated with 2′-C-methyladenosine. (PDF) [file ppat.1003030.s004.pdf]

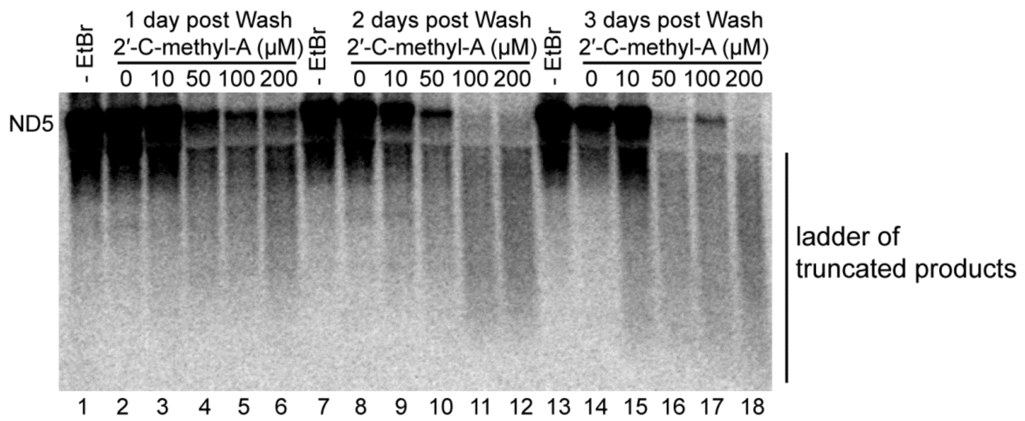

Figure S4. Production of Full-Length Mitochondrial RNA Transcripts is Impaired in the Presence of 2′-C-methyladenosine: Overexposed Northern Blots Showing a Ladder of Truncated Products. Northern blot of ND5 after EtBr treatment and recovery in the presence of 2′-C-methyladenosine. The blot was overexposed and shows a ladder of truncated RNA products that accumulate over time from cells treated with 2′-C-methyladenosine.
