# Supplementary material for: Sensitivity of Mitochondrial Transcription and Resistance of RNA Polymerase II Dependent Nuclear Transcription to Antiviral Ribonucleosides
Source: PLoS Pathog. 2012 Nov 15;8(11):e1003030. doi: 10.1371/journal.ppat.1003030 (PMC3499576; doi:10.1371/journal.ppat.1003030)
Supplement: Figure S5 — Kinetics of TFIIS-mediated RNA cleavage. (A,B) Kinetics of TFIIS-mediated RNA cleavage subsequent to AMP, 2′C-methyl-AMP and 3′-dAMP incorporation by calf thymus Pol II. TEC-C12 was incubated with 500 µM ATP, 2′-C-methyl-ATP or 3′-dATP for 2 min at which point TFIIS added to 1 µM final concentration for the indicated time. The percentage of A13 remaining was plotted as a function of time and fit to a single exponential yielding observed rate constants of TFIIS-mediated RNA cleavage of 0.040±0.006, 0.0060±0.0020, 0.14±0.02 s−1 after incorporation of AMP, 2′-C-methyl-AMP or 3′-dAMP respectively. (C,D) Kinetics of TFIIS-mediated RNA cleavage subsequent to CMP, 2′C-methyl-CMP and 4′-azido-CMP incorporation. TEC-A11 was incubated with 500 µM CTP or 2′-C-methyl-CTP for 2 min or 4′-azido-CTP for 10 min at which point TFIIS added to 1 or 10 µM final concentration for the indicated time. The percentage of C12 remaining was plotted as a function of time and fit to a single exponential yielding observed rate constants of TFIIS-mediated RNA cleavage of 0.088±0.003, 0.0018±0.0003, 0.13±0.01 and 0.13±0.01 s−1 after incorporation of CMP, 2′-C-methyl-CMP or4′-azido-CMP respectively. At 10 µM TFIIS the observed rate constant of TFIIS-mediated RNA cleavage after incorporation of 2′-C-methyl-CMP was 0.0045±0.0004 s−1. (PDF) [file ppat.1003030.s005.pdf]

Fig. S5

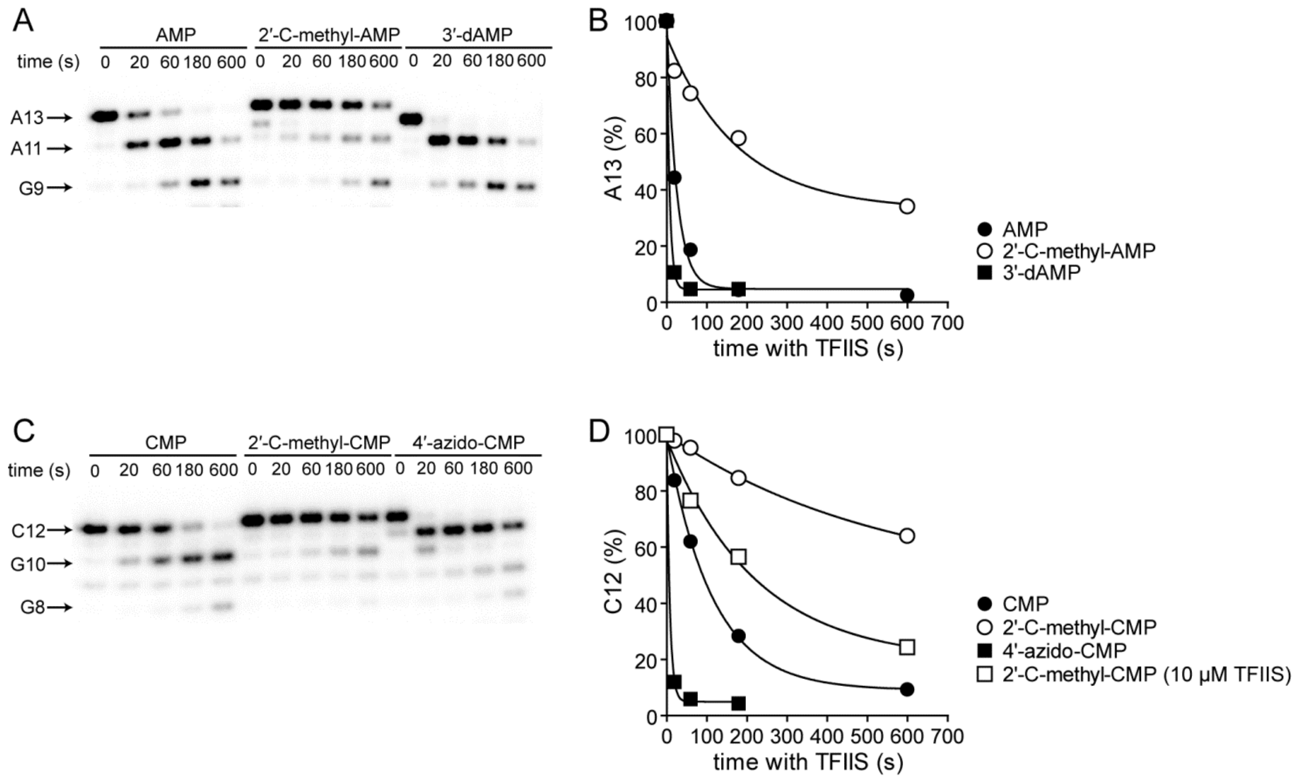

Figure S5. Kinetics of TFIIS-Mediated RNA Cleavage. (**A,B**) Kinetics of TFIIS-mediated RNA cleavage subsequent to AMP, 2′C-methyl-AMP and 3′-dAMP incorporation by calf thymus Pol II. TEC-C12 was incubated with 500 μM ATP, 2′-C-methyl-ATP or 3′-dATP for 2 min at which point TFIIS added to 1 μM final concentration for the indicated time. The percentage of A13 remaining was plotted as a function of time and fit to a single exponential yielding observed rate constants of TFIIS-mediated RNA cleavage of 0.040 ± 0.006, 0.0060 ± 0.0020, 0.14 ± 0.02 s^{-1} after incorporation of AMP, 2′-C-methyl-AMP or 3′-dAMP respectively. (**C,D**) Kinetics of TFIIS-mediated RNA cleavage subsequent to CMP, 2′C-methyl-CMP and 4′-azido-CMP incorporation. TEC-A11 was incubated with 500 μM CTP or 2′-C-methyl-CTP for 2 min or 4′-azido-CTP for 10 min at which point TFIIS added to 1 or 10 μM final concentration for the indicated time. The percentage of C12 remaining was plotted as a function of time and fit to a single exponential yielding observed rate constants of TFIIS-mediated RNA cleavage of 0.088 ± 0.003, 0.0018 ± 0.0003, 0.13 ± 0.01 and 0.13 ± 0.01 s^{-1} after incorporation of CMP, 2′-C-methyl-CMP or4′-azido-CMP respectively. At 10 μM TFIIS the observed rate constant of TFIIS-mediated RNA cleavage after incorporation of 2′-C-methyl-CMP was 0.0045 ± 0.0004 s^{-1}.
