# Supplementary material for: Sensitivity of Mitochondrial Transcription and Resistance of RNA Polymerase II Dependent Nuclear Transcription to Antiviral Ribonucleosides
Source: PLoS Pathog. 2012 Nov 15;8(11):e1003030. doi: 10.1371/journal.ppat.1003030 (PMC3499576; doi:10.1371/journal.ppat.1003030)
Supplement: Figure S6 — Nucleoside analog incorporation and extension of the resulting products catalyzed by yeast Pol II. (A) Schematic of synthetic nucleic scaffolds for transcription elongation complex (TECs) assembly with yeast Pol II. The TECs were assembled using TDS76 and NDS79 and either RNA9 in the presence of 10 µM GTP (TEC-G10) or RNA 7(20) in the presence of 10 µM each ATP, GTP and UTP (TEC-A11); the TECs were purified from the unincorporated DNA, RNA and NTPs before addition of the appropriate nucleoside analog triphosphate. The oligonucleotide sequences are in Table S2. (B,C) Reaction products from Pol II-catalyzed nucleotide incorporation in the absence and presence of TFIIS. The concentration of the unmodified substrate NTP and analogs were 500 µM; TFIIS was added at 10 µM. Reactions proceeded for 1 min. Reaction with ribavirin-TP proceeded for 10 min. (D,E) Reaction products from Pol II-catalyzed nucleotide incorporation in the presence of the next correct nucleotide substrate. The concentration of the unmodified substrate NTP and analogs were 500 µM; TFIIS was added at 10 µM. Reactions proceeded for 1 min. Reaction with ribavirin-TP proceeded for 10 min. (F) Percent inhibition by TFIIS on Pol II nucleoside analog incorporation. (PDF) [file ppat.1003030.s006.pdf]

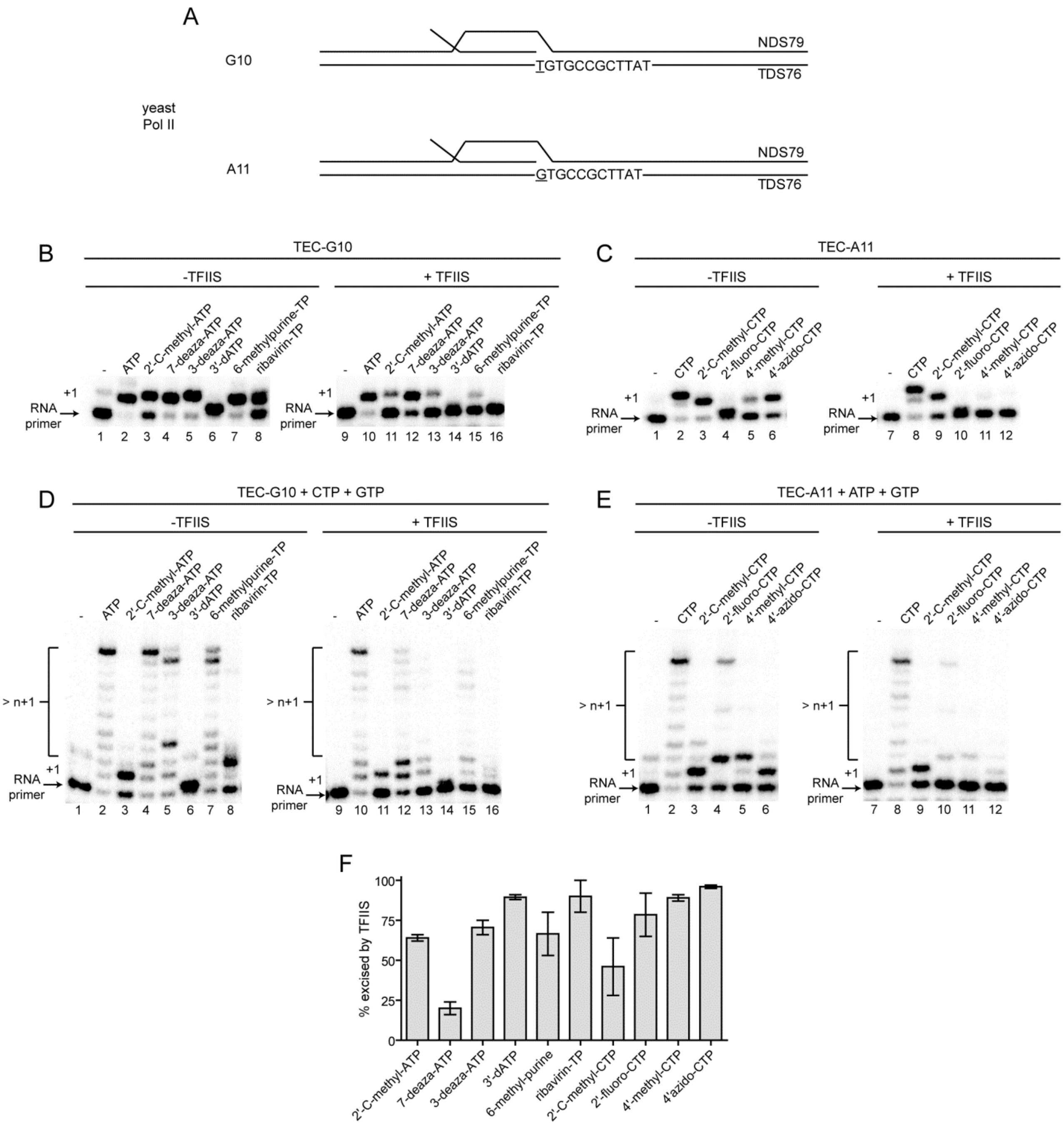

Figure S6. Nucleoside Analog Incorporation and Extension of the Resulting Products Catalyzed by Yeast Pol II. (**A**) Schematic of synthetic nucleic scaffolds for transcription elongation complex (TECs) assembly with yeast Pol II. The TECs were assembled using TDS76 and NDS79 and either RNA9 in the presence of 10 μM GTP (TEC-G10) or RNA 7(20) in the presence of 10 μM each ATP, GTP and UTP (TEC-A11); the TECs were purified from the unincorporated DNA, RNA and NTPs before addition of the appropriate nucleoside analog triphosphate. The oligonucleotide sequences are in Table S2. (**B,C**) Reaction products from Pol II-catalyzed nucleotide incorporation in the absence and presence of TFIIS. The concentration of the unmodified substrate NTP and analogs were 500 μM; TFIIS was added at 10 μM. Reactions proceeded for 1 min. Reaction with ribavirin-TP proceeded for 10 min. (**D,E**) Reaction products from Pol II-catalyzed nucleotide incorporation in the presence of the next correct nucleotide substrate. The concentration of the unmodified substrate NTP and analogs were 500 μM; TFIIS was added at 10 μM. Reactions proceeded for 1 min. Reaction with ribavirin-TP proceeded for 10 min. (**F**) Percent inhibition by TFIIS on Pol II nucleoside analog incorporation.
