# Supplementary material for: Sensitivity of Mitochondrial Transcription and Resistance of RNA Polymerase II Dependent Nuclear Transcription to Antiviral Ribonucleosides
Source: PLoS Pathog. 2012 Nov 15;8(11):e1003030. doi: 10.1371/journal.ppat.1003030 (PMC3499576; doi:10.1371/journal.ppat.1003030)
Supplement: Protocol S1 — Polymerase-catalyzed nucleotide incorporation assays. (DOCX) [file ppat.1003030.s007.docx]

**Protocol S1. Polymerase-Catalyzed Nucleotide Incorporation Assays.**

**Human Mitochondrial RNA Polymerase Nucleotide Incorporation Assays***.* Nucleotide incorporation experiments were performed as described previously [1]. In general, elongation complexes were formed by incubating POLRMT with the appropriate primed template followed by rapid mixing with nucleoside triphosphate substrate in reaction buffer consisting of 1X MTCN (50 mM MES, 25 mM TRIS, 25 mM CAPS and 50 mM NaCl) pH 7.5, 10 mM MgCl_2_ and 1 mM DTT. All reactions were performed at 30 ^o^C. For all reactions in this study with a final NTP concentration higher than 1 mM, the amount of free Mg^2+^ was kept constant by increasing the amount of MgCl_2_ in the reaction to 10 mM plus the NTP concentration above 1 mM. POLRMT was diluted immediately prior to use in enzyme dilution buffer (EDB) which consisted of 10 mM Tris pH 8.0, 1 mM DTT and 20% glycerol. POLRMT of comparable activity and purity can be obtained from Indigo Biosciences, Inc. (State College, PA). The volume of POLRMT added to any reaction was always less than or equal to one-tenth the total volume. For single nucleotide incorporation experiments, purified POLRMT (0.5 µM) was assembled onto ^32^P-labeled RNA primer/DNA template nucleic acid scaffold (0.25 µM) for 1 min, nucleoside triphosphate substrate (500 µM) was added, reactions proceeded for 30 s and quenching was by addition of EDTA (50 mM). For chain termination experiments, POLRMT (0.5 µM) was assembled onto a ^32^P-labeled RNA primer/DNA template nucleic acid scaffold (0.25 µM) for 1 min, nucleoside triphosphate substrate (500 µM) was added in the presence of the next correct nucleotide, UTP or ATP (50 µM), reactions proceeded for 10 min and quenching was by addition of EDTA (50 mM). Products were resolved from substrates by denaturing PAGE. An equal volume of loading buffer, 5 µL, (70% formamide, 0.025% bromophenol blue and 0.025% xylene cyanol) was added to 5 µL of quenched reaction mixtures and heated to 70 °C for 2-5 min prior to loading 5 µL on a denaturing 23% polyacrylamide gel containing 1X TBE (89 mM Tris base, 89 mM boric acid, 2 mM EDTA) and 7 M Urea. Electrophoresis was performed in 1X TBE at 90 W. Gels were visualized by using a PhosphorImager (GE) and quantified by using ImageQuant software (GE).

**Rapid Chemical-Quench-Flow Experiments.** Rapid mixing/quenching experiments were performed by using a Model RQF-3 chemical-quench-flow apparatus (KinTek Corp., Austin, TX) equipped with a water bath. All reactions were performed at 30 °C. Reactions were performed by incubating POLRMT (0.25 µM) with ^32^P-labeled RNA primer/DNA template nucleic acid scaffold (0.25 µM) at room temperature for 3 min, equilibrated to 30 °C and then rapidly mixed with various concentrations of nucleoside triphosphate substrate. Reactions were quenched by addition of EDTA (0.3 M). Products were resolved from substrates by denaturing PAGE.

**Stopped-Flow Fluorescence Assays.** Stopped-flow assays were performed using a Model SF-2001 stopped-flow apparatus (Kintek Corp., Austin, TX) equipped with a water-bath. All reactions were at 30 °C. Reactions were performed by incubating POLRMT (0.25 µM) with RNA primer/DNA template nucleic acid scaffold having a DNA template with 2-aminopurine (2AP) at the +1 templating position[1,2] at room temperature for 3 minutes, equilibrated to 30 °C and then rapidly mixed with various concentrations of nucleoside triphosphate substrate. The excitation wavelength was 313 nm. Fluorescence emission was monitored by using a 370 nm cut-on filter (model E370LP, Chroma technology corp., Rockingham, VT). For each experiment, at least four fluorescence traces were averaged.

**Determination of Kinetic Parameters *K*_d,app_ and *k*_pol._** Kinetic data were fit by nonlinear regression using the program KaleidaGraph (Synergy Software, Reading, PA). Observed rate constants (*k*_obs_) for nucleotidyl transfer at various concentrations of nucleotide substrate were obtained by fitting either product-versus-time data or relative fluorescence-versus-time data to an equation defining a single exponential (Eq. 1), where A is the amplitude, *k*_obs_ is the observed rate constant and C is the endpoint.

$$F=A*e \left( -kobs*t \right)+ C$$

Values for *k*_pol_, the maximum rate constant for single-nucleotide incorporation, and *K*_d,app_, the apparent dissociation constant for NTP were obtained by fitting *k*_obs_-versus-[NTP] data to an equation defining a hyperbola (Eq. 2).

$$kobs=\frac{kpol*[NTP]}{Kd,app+[NTP]}$$

**Pol II Elongation Complex Assembly and Transcription Elongation Assays***.* Human recombinant TFIIS was expressed and purified as described in [3]. Yeast TFIIS was expressed and purified according to [4] using expression construct generously provided by C. Kane. Elongation complexes assembly with yeast RNA polymerase II and their purification using centrifugal ultrafiltration is described in detail in [5,6]. Elongation complexes assembly with bovine Pol II and their purification were performed in the same conditions as with yeast Pol II. Transcription by bovine and yeast Pol II was done at 25 °C and 37 °C, respectively, in 20 mM Tris-HCl, pH 7.9, 40 mM KCl, 5 mM MgCl_2_, 10 µM ZnCl_2_, 5 mM β-mercaptoethanol.

#### HCV NS5B Enzymatic Assays. HCV NS5B-dependent RNA elongation activity was assayed using a heteropolymeric RNA template described previously [7]. All concentrations refer to the final concentrations unless mentioned otherwise. A reaction mixture containing 50 mM Tris-HCl (pH 7.5), 10 mM KCl, 1 mM DTT, 0.1 mg/mL BSA, 0.2 unit/µL RNasin Plus RNase Inhibitor (Promega, Madison, WI, cat # N261B), 4 ng/µL sshRNA, 5 mM MgCl_2_, and 70 nM HCV NS5B (con 1b delta 21) was pre-incubated with NTP analogs for 5 min at room temperature. The reaction was initiated by the addition of a mixture containing 2.5 µM ATP, 2.5 µM CTP, 2.5 µM UTP, 1.25 µM GTP, 0.06 µCi/µL of α-^33^P-GTP (3000 mCi/mol). Reactions were allowed to proceed for 90 minutes at 30 °C and then transferred onto 96-well DE81 filter membranes, washed with 100 mM Na_2_HPO_4_ (3x), ethanol (1x), and air dried. Scintillation fluid was added to the wells and counts per minute (cpm) were measured using a TopCount instrument (Perkin Elmer, Waltham, MA). IC_50_ values were determined using Prism 4.0 software (GraphPad, San Diego, CA).

**References**

1. Smidansky ED, Arnold JJ, Reynolds SL, Cameron CE (2011) Human mitochondrial RNA polymerase: evaluation of the single-nucleotide-addition cycle on synthetic RNA/DNA scaffolds. Biochemistry 50: 5016-5032.

2. Castro C, Smidansky E, Maksimchuk KR, Arnold JJ, Korneeva VS, et al. (2007) Two proton transfers in the transition state for nucleotidyl transfer catalyzed by RNA- and DNA-dependent RNA and DNA polymerases. Proc Natl Acad Sci U S A 104: 4267-4272.

3. Yoo OJ, Yoon HS, Baek KH, Jeon CJ, Miyamoto K, et al. (1991) Cloning, expression and characterization of the human transcription elongation factor, TFIIS. Nucleic Acids Res 19: 1073-1079.

4. Awrey DE, Weilbaecher RG, Hemming SA, Orlicky SM, Kane CM, et al. (1997) Transcription elongation through DNA arrest sites. A multistep process involving both RNA polymerase II subunit RPB9 and TFIIS. J Biol Chem 272: 14747-14754.

5. Kireeva M, Nedialkov YA, Gong XQ, Zhang C, Xiong Y, et al. (2009) Millisecond phase kinetic analysis of elongation catalyzed by human, yeast, and Escherichia coli RNA polymerase. Methods 48: 333-345.

6. Kireeva ML, Domecq C, Coulombe B, Burton ZF, Kashlev M (2011) Interaction of RNA polymerase II fork loop 2 with downstream non-template DNA regulates transcription elongation. J Biol Chem 286: 30898-30910.

7. Paulson MS, Yang H, Shih IH, Feng JY, Mabery EM, et al. (2009) Comparison of HCV NS3 protease and NS5B polymerase inhibitor activity in 1a, 1b and 2a replicons and 2a infectious virus. Antiviral Res 83: 135-142.
