# Supplementary material for: Sensitivity of Mitochondrial Transcription and Resistance of RNA Polymerase II Dependent Nuclear Transcription to Antiviral Ribonucleosides
Source: PLoS Pathog. 2012 Nov 15;8(11):e1003030. doi: 10.1371/journal.ppat.1003030 (PMC3499576; doi:10.1371/journal.ppat.1003030)
Supplement: Protocol S2 — Evaluation of nucleoside analogs in cells. (DOCX) [file ppat.1003030.s008.docx]

**Protocol S2. Evaluation of Nucleoside Analogs in Cells.**

**Isolation of Mitochondria.** HeLa S3 cells were seeded in spinner flasks at a density of 1 x 10^6^ cells per mL in a final volume of 100 mL. Cells were incubated with compounds (10 µM each, 2′-C-methyladenosine or cordycepin) for 24 h at 37 °C in a 5% CO_2_ incubator. After treatment, cells were harvested, washed two times with ice cold PBS, placed on ice and resuspended in hypotonic buffer (3.5 mM Tris HCl, pH 7.8, 2.5 mM NaCl, 0.5 mM MgCl_2_). Cells were homogenized using dounce homogenizer and made isotonic by adding 0.35 M Tris-HCl, pH 7.8, 0.25 M NaCl, 50 mM MgCl_2_. This homogenate was centrifuged at 1,200 × *g* for 3 min at 4 °C. The supernatant obtained was used to pellet mitochondria by centrifugation at 15,000 × *g* for 2 min at 4 °C. This mitochondrial pellet was washed with buffer B (1 mM Tris-HCl (pH 7.0), 0.13 M NaCl. 5 mM KCl, 7.5 mM MgCl_2_) twice and resuspended in buffer C (10 mM Tris-HCl (pH 7.4), 1 mM EDTA. Mitochondria were frozen on dry ice and stored in -80 ºC until further analysis. Mitochondria from untreated cells served as controls.

**Intracellular Metabolism.** Huh-7 cells were transferred to 12-well tissue culture treated plates by tryposonization and grown to confluency (0.88 x 10^6^ cells/well). For each independent experiment, duplicate wells were respectively treated with 10 µM of each ribonucleoside analog. Triphosphate levels had to be dose normalized after being determined with incubations at 0.1 µM 7-deazaadenosine and 6-methylpurine due to toxicity observed at 10 µM. After 24 hours, wells were washed twice with 5 mL of ice cold normal saline (0.9% w/v NaCl) prior to cell samples being harvested by scraping into 1 mL of 70% methanol. MT4 cells were passaged into T10 flasks at 0.5 x 10^6^/mL (final volume 5 mL) and grown overnight prior to the start of the experiment. For each independent experiment, duplicate flasks were respectively treated with 10 µM of each ribonucleoside analogs. Triphosphate levels had to be dose normalized after being determined with incubations at 0.1 µM 7-deazaadenosine, 6-methylpurine and 2′-deoxy-2′-fluorocytidine due to toxicity observed at 10 µM. After 24 hours, cells were counted and isolated by spinning through oil essentially as previously described [1], except that the oil layer was washed with ice cold normal saline instead of phosphate buffered saline.

**Analysis of Nucleotide Concentrations by Liquid Chromatography Coupled to Tandem Mass Spectrometry (LC/MS/MS).** After storing samples in 70% methanol at -20 °C for at least overnight to facilitate cell lysis, cell (Huh-7 and MT4) or mitochondrial (from HeLa S3 cells) lysates were dried and resuspended in 3 mM ammonium formate (pH 5) with 10 mM dimethylhexylamine in water at a concentration of extract from 100,000 cells/10 µL for Huh-7 and MT4 cells or extract from mitochondria isolated from 1 million cells/10 µL for mitochondria isolated from HeLa S3 cells and 10 µL of the samples were analyzed by LC/MS/MS. Ribonucleoside triphosphates were quantified by ion pairing LC coupled to positive mode MS/MS essentially as described previously ([2]. Briefly, dimethylhexylamine was used as an ion pairing agent and the mass to charge (m/z) transitions of 585.1/97.1, 492.1/136.1, 207.1/135.1, 507.2/135.1, 507.0/135.1, 522.2/136.1, 418.2/112.1, 406.2/112.1, 498/112 and 525/112 were used for ribavirin-TP, cordycepin-TP, 6-methylpurine-ribonucleoside-TP, 7-deaza-ATP, 3-deaza-ATP, 2′-C-methyl-ATP, 2′-C-methyl-CTP, 2′-deoxy-2′-fluoro-CTP, 4′-C-methyl-CTP and 4′-azido-CTP, respectively, for MS/MS detection. Endogenous ATP in isolated mitochondria was quantified using the LC/MS/MS methods described above and standard curves of stable isotopically labeled ATP as described previously[3]. 2-chloro-ATP (m/z transition of 542.2/170.1) was added as an internal standard to all samples. Seven point standard curves, used to quantify each nucleotide, covered at least three orders of magnitude and had linearity exceeding an r^2^ value of 0.98. To ensure accuracy and precision within 20% over the course of the analyses, quality control samples containing each nucleotide being studied were injected at the beginning and end of each analytical batch. Intracellular concentrations for Huh-7 and MT4 cells were converted from pmol of nucleotide per million cells to an intracellular molar concentration using a cellular volume of 2 pL per cell. Molar concentrations in mitochondria isolated from HeLa S3 cells were estimated based upon that 10% of the total cellular volume is accounted for by mitochondria[4].

**Cytotoxicity Assays.** 5-day cytotoxicity assay in Huh-7 cells: The 5-day cytotoxicity of compounds was tested in Huh-7 cells using CellTiter Glo viability reagents (Promega). Three-fold serial dilutions of compounds were prepared in duplicate in 96-well plates starting at a top concentration of 100 μM. Huh-7 cells were seeded at density of 3.0 × 10^3^ cells /well. After cells attached, serially diluted compounds were added in a final volume of 200 μL per well with constant 0.5% DMSO. The cells were incubated with compounds for five days in 37º C incubator, the culture medium was removed before adding 100 μL /well of CellTiter Glo viability reagents. The compound’s cytotoxicity was determined by measuring luminescence (ATP level) on a Victor Luminescence plate reader (Perkin-Elmer). CC_50_ values were calculated using Dose Response Analysis. 5-day cytotoxicity assay in MT4 cells: The 5-day cytotoxicity of compounds was tested in MT4 cells using CellTiter Glo viability reagents (Promega). Three-fold serial dilutions of compounds were prepared in duplicate in 384-well plates starting at a concentration of 100 μM. MT-4 cells were plated at a density of 2 × 10^3^ cells per well in a final assay volume of 40 μL per well with a constant amount of DMSO equal to 0.5%. Cells were incubated with compounds for five days at 37 ºC in a 5% CO_2_ incubator. Cell viability was determined by adding 40 μL per well of CellTiter Glo viability reagents (Promega, Madison, WI). After incubation for 10-30 minutes at room temperature the luminescence signal was quantified on a Victor Luminescence plate reader (Perkin-Elmer).

**Determination of CC_50_ Values.** In cell viability assays, the CC_50_ value is defined as the concentration at which there was a measured 50% decrease in cellular ATP level. Data was analyzed using Pipeline Pilot software (Accelrys, San Diego, CA) or GraphPad Prism 5.0 (La Jolla, CA). Unless otherwise mentioned, CC_50_ values were calculated by non-linear regression analysis using sigmoidal dose-response (variable slope) equation (four parameter logistic equation) (Eq. 3) where the Bottom and Top values were fixed at 0 and 100, respectively. CC_50_ values were calculated as an average of two to four independent experiments.

$$\% viability= {Bottom+(Top-Bottom)}/{(1+10^\left( (LogCC50-[Nucleoside] \right)*HillSlope))}$$

**HCV Replicon Assays.** A Huh-7 cell line carrying a Renilla luciferase HCV genotype 1b replicon was used to assess antiviral activity of compounds as described previously [5,6]. Briefly, replicon cells were seeded in 384-well plates at a density of 1.8 × 10^3^ cells per well in 90 μL of DMEM culture medium supplemented with 10% FBS. Compounds were serially diluted in 100% DMSO and added to cells at a 1:225 dilution, achieving a final concentration of 0.44% DMSO. Cell plates were incubated at 37 °C for 3 days, after which culture medium was removed, and cells were washed in 1X PBS to remove residual media using a Biotek ELX405 plate washer. A volume of 20 μL of Dual-Glo luciferase buffer (Promega, Madison, WI, Cat#E298B) was added to each well of the plate with a Biotek µFlow Workstation. The plate was incubated for 10 minutes at room temperature. A volume of 20 μL of a solution containing 1:100 mixture of Dual-Glo Stop & Glo substrate (Promega, Madison, WI, Cat#E313B) and Dual-Glo Stop & Glo buffer (Promega, Madison, WI, Cat#E314B) was then added to each well of the plate with a Biotek μFlow Workstation. The plate was then incubated at room temperature for 10 minutes before the luminescence signal was measured with a Perkin Elmer Envision Plate Reader.

**Determination of EC_50_ Values.** The percent inhibition of HCV replicon was calculated based on positive control (0.5 μM ITMN-191 treated cells) and negative control (0.44% DMSO treated cells). EC_50_ values were determined by nonlinear regression using Pipeline Pilot (Accelrys, San Diego, CA).

**Mitochondrial Transcription in Cells.** Huh7 cells were seeded at a density of 0.5 x 10^6^ cells. Once cells attached, cells were treated with 50 ng/mL ethidium bromide and placed at 37 °C in a 5% CO_2_ incubator to deplete mitochondrial transcripts from cells. After 24 h, media was removed, cells were washed with PBS and fresh media was added containing the appropriate compound at various concentrations. DMSO at 0.5% was used as a negative control. Cells were placed at 37 °C in a 5% CO_2_ incubator for one, two or three days. Every 24 h the media was removed and replaced with fresh media containing the appropriate compound. At the indicated time point, media was removed, cells washed with PBS, harvested and Northern blots performed.

**Northern Blot Analysis.** Northern blots were performed for the detection of mitochondrial transcripts ND1 and ND5 and nuclear transcript GAPDH. Cells were lysed by adding TRI Reagent (Sigma, Cat# T94247) with 0.2 mL of chloroform added per 1 mL of TRI Reagent. Samples were vigorously shaken by hand for 15 seconds and incubated at room temperature for 2 to 3 minutes followed by centrifugation at 12,000 × *g* for 15 minutes at 4°C. RNA from the aqueous phase was precipitated by mixing with (0.5 mL) isopropyl alcohol. Samples were incubated at room temperature for 10 minutes and then centrifuged at 12,000 × *g* for 10 minutes at 4°C. NA pellets were washed with 75% ethanol and centrifuged at 7,500 × *g* for 5 minutes at 4°C. RNA pellets finally dissolved in RNase-free water. The RNA was separated on a 1% denaturing agarose gel (0.8 M formaldehyde in 1× MOPS) by running at 120V for 2 h until the bromophenol blue band migrated through three-fourths of the gel. The gel was washed in water for 30 min twice followed by soaking in 20× SSC (1X SSC: 0.15 M NaCl and 0.015 M sodium citrate) for 30 min. RNA was transferred to nylon membrane (Hybond XL, GE Healthcare) using capillary blotting with 20× SSC for 16 h at room temperature. The membrane was slightly dried and RNA was crosslinked to the membrane using a UV Crosslinker (Stratalinker 2400, Stratagene). The membrane was washed twice with wash buffer (1× SSC and 0.1% SDS) at 65 °C for 30 min each. The membrane was prehybridized in 100 mL of modified Church’s buffer (0.5 M sodium phosphate, pH 7.2, 7% SDS, and 1 mM EDTA) for 4 h at 65 °C. The hybridization probe was denatured for 5 min at 95 °C and chilled on ice for 1 min. Hybridization was performed in a modified Church’s buffer for 16 h at 65 °C. The membrane was washed in wash buffer (1× SSC and 0.1% SDS) for 20 min at 65 °C twice and once at room temperature. RNA was visualized by exposing the membrane to a phosphor screen followed by scanning the screen on a Typhoon 8600 scanner (Promega) in phosphor mode. Hybridization probes were made by PCR using DNA oligos and mtDNA or nuclear DNA as a template. Probes for GAPDH were used as a positive control. Probe sequences are as follows: ND1-for, 5′-GCCAACCTCCTACTC-3′; ND1-rev, 5′-GAGGGGGAATGCTGG-3′; ND5-for, 5′-GCAGATGCCAACACA-3′; ND5-rev, 5′-GCTTCCGGCTGCTAG-3′; GAPDH-for, 5′-TGTGGTCATGAGTCCTTCCA-3′; GAPDH-rev, 5′- CGAGATCCCTCCAAAATCAA-3′. In the PCR reaction, [α-^32^P]-dATP (1 mCi/mL, 3000 Ci/mmol, GE Healthcare) was used with cold dNTPs (300 µM for each dCTP, dGTP, and dTTP and 10 µM for dATP) in total volume of 100 µL per reaction. The quality of PCR product was confirmed by agarose gel electrophoresis and cpm was determined by using scintillation counter (LKB Wallac 1217 Rackbeta liquid scintillation counter).

**References**

1. Ray AS, Olson L, Fridland A (2004) Role of purine nucleoside phosphorylase in interactions between 2',3'-dideoxyinosine and allopurinol, ganciclovir, or tenofovir. Antimicrob Agents Chemother 48: 1089-1095.

2. Durand-Gasselin L, Van Rompay KK, Vela JE, Henne IN, Lee WA, et al. (2009) Nucleotide analogue prodrug tenofovir disoproxil enhances lymphoid cell loading following oral administration in monkeys. Mol Pharm 6: 1145-1151.

3. Vela JE, Miller MD, Rhodes GR, Ray AS (2008) Effect of nucleoside and nucleotide reverse transcriptase inhibitors of HIV on endogenous nucleotide pools. Antivir Ther 13: 789-797.

4. Schon EA, Pon LA (2001) Mitochondria, Methods in Cell Biology: Academic Press.

5. Robinson M, Tian Y, Pagratis N, Delaney WE (2011) Screening of hepatitis C virus inhibitors using genotype 1a HCV replicon cell lines. Curr Protoc Microbiol Chapter 17: Unit17 17.

6. Robinson M, Yang H, Sun SC, Peng B, Tian Y, et al. (2010) Novel hepatitis C virus reporter replicon cell lines enable efficient antiviral screening against genotype 1a. Antimicrob Agents Chemother 54: 3099-3106.
