# Supplementary material for: Sensitivity of Mitochondrial Transcription and Resistance of RNA Polymerase II Dependent Nuclear Transcription to Antiviral Ribonucleosides
Source: PLoS Pathog. 2012 Nov 15;8(11):e1003030. doi: 10.1371/journal.ppat.1003030 (PMC3499576; doi:10.1371/journal.ppat.1003030)
Supplement: Table S1 — Mitochondrial ribonucleoside triphosphate concentrations. (DOCX) [file ppat.1003030.s009.docx]

**Table S1.** **Mitochondrial Ribonucleoside Triphosphate Concentrations.**

| **Ribonucleoside triphosphate** | **Concentration***^a^* **(µM)** |
| --- | --- |
| ATP*^b^* | 8,600 ± 400^b^ |
| CTP*^b^* | 250 ± 20 |
| GTP*^b^* | 2,400 ± 180 |
| UTP*^b^* | 1,800 ± 1,600 |
| 2′-C-methyl-ATP*^c^* | 210 ± 40 |
| 3′-dATP*^c^* | < 30 |

*^a^*Concentrations calculated assuming that mitochondria account for 10% of the total cellular volume.

*^b^*Values are the mean ± standard deviation from three independent mitochondrial isolations.

*^c^*Concentrations determined after 24 h incubations with 100 µM of 2′-C-methyladenosine and 3′-deoxyadenosine nucleoside.
