# Supplementary material for: Sensitivity of Mitochondrial Transcription and Resistance of RNA Polymerase II Dependent Nuclear Transcription to Antiviral Ribonucleosides
Source: PLoS Pathog. 2012 Nov 15;8(11):e1003030. doi: 10.1371/journal.ppat.1003030 (PMC3499576; doi:10.1371/journal.ppat.1003030)
Supplement: Table S2 — Oligonucleotides used for the elongation complex assembly. (DOCX) [file ppat.1003030.s010.docx]

**Table S2. Oligonucleotides Used for the Elongation Complex Assembly.**

| **Oligo** | **Sequence** |
| --- | --- |
| NDS79 | 5′CCTATAGGATACTTACAGCCATCGAGAGGGACACGGCGAATAGCCATCCCAATCCACACGTCCAACGGGGCAAACCGTA3′ |
| TDS76 | 5′GGTTTGCCCCGTTGGACGTGTGGATTGGGATGGCTATTCGCCGTGTCCCTCTCGATGGCTGTAAGTATCCTATAGG3′ |
| NDS50 | 5′GGTATAGGATACTTACAGCCATCGAGAGGGACATTTCGAAAAGAGAACCC3′ |
| TDS50 | 5′GGGTTCTCTTTTCGAAATGTCCCTCTCGATGGCTGTAAGTATCCTATACC3′ |
| RNA7(20) | 5′CGGCGAA3′ |
| RNA9 | 5′AUCGAGAGG3′ |
